# The endocytic recycling compartment serves as a viral factory for hepatitis E virus

**DOI:** 10.1007/s00018-022-04646-y

**Published:** 2022-12-03

**Authors:** Cyrine Bentaleb, Kévin Hervouet, Claire Montpellier, Charline Camuzet, Martin Ferrié, Julien Burlaud-Gaillard, Stéphane Bressanelli, Karoline Metzger, Elisabeth Werkmeister, Maliki Ankavay, Nancy Leon Janampa, Julien Marlet, Julien Roux, Clarence Deffaud, Anne Goffard, Yves Rouillé, Jean Dubuisson, Philippe Roingeard, Cécile-Marie Aliouat-Denis, Laurence Cocquerel

**Affiliations:** 1 University of Lille, CNRS, Inserm, CHU Lille, Pasteur Institute of Lille, U1019-UMR 9017-CIIL-Center for Infection and Immunity of Lille, 59000 Lille, France; 2 Inserm U1259, Morphogénèse et Antigénicité du VIH et des Virus des Hépatites (MAVIVH), Université de Tours and CHRU de Tours, 37032 Tours, France; 3 Université de Tours et CHRU de Tours, Plateforme IBiSA de Microscopie Electronique, Tours, France; 4 Université Paris-Saclay, CEA, CNRS, Institute for Integrative Biology of the Cell (I2BC), Gif-Sur-Yvette, France; 5 Univ. Lille, CNRS, Inserm, CHU Lille, Institut Pasteur de Lille, UMR2014-US41-PLBS-Plateformes Lilloises de Biologie and Santé, Lille, France; 6 BIOTEM, Apprieu, France; 7 Present Address: Division of Gastroenterology and Hepatology, Institute of Microbiology, Lausanne, Switzerland

**Keywords:** Hepatitis E virus, ORF2 capsid protein, Antibodies, Infectious particles, AlphaFold2, Viral factories, Electron microscopy, Endocytic recycling compartment, Rab11

## Abstract

Although hepatitis E virus (HEV) is the major leading cause of enterically transmitted viral hepatitis worldwide, many gaps remain in the understanding of the HEV lifecycle. Notably, viral factories induced by HEV have not been documented yet, and it is currently unknown whether HEV infection leads to cellular membrane modeling as many positive-strand RNA viruses. HEV genome encodes the ORF1 replicase, the ORF2 capsid protein and the ORF3 protein involved in virion egress. Previously, we demonstrated that HEV produces different ORF2 isoforms including the virion-associated ORF2i form. Here, we generated monoclonal antibodies that specifically recognize the ORF2i form and antibodies that recognize the different ORF2 isoforms. One antibody, named P1H1 and targeting the ORF2i N-terminus, recognized delipidated HEV particles from cell culture and patient sera. Importantly, AlphaFold2 modeling demonstrated that the P1H1 epitope is exposed on HEV particles. Next, antibodies were used to probe viral factories in HEV-producing/infected cells. By confocal microscopy, we identified subcellular nugget-like structures enriched in ORF1, ORF2 and ORF3 proteins and viral RNA. Electron microscopy analyses revealed an unprecedented HEV-induced membrane network containing tubular and vesicular structures. We showed that these structures are dependent on ORF2i capsid protein assembly and ORF3 expression. An extensive colocalization study of viral proteins with subcellular markers, and silencing experiments demonstrated that these structures are derived from the endocytic recycling compartment (ERC) for which Rab11 is a central player. Hence, HEV hijacks the ERC and forms a membrane network of vesicular and tubular structures that might be the hallmark of HEV infection.

**Supplementary Information:**

The online version contains supplementary material available at 10.1007/s00018-022-04646-y.

## Introduction

Hepatitis E virus (HEV) is the most common cause of acute viral hepatitis worldwide. Five distinct genotypes (gt), belonging to a single serotype, infect humans. HEV gt1 and gt2 are restricted to humans and are responsible for waterborne outbreaks in developing countries with low sanitary conditions. HEV gt3, gt4 and gt7 are zoonotic and cause sporadic zoonotic foodborne hepatitis in industrialized countries and United Arab Emirates [1–4]. Although HEV causes a mostly asymptomatic self-limited disease, gt1-infection can lead to fulminant liver failure, particularly in pregnant women, and gt3-infection can lead to chronic disease in immunosuppressed patients. There is no specific treatment nor universal vaccine against HEV [5].

HEV is found as a non-enveloped virus in bile and feces or as a quasi-enveloped virus (eHEV) in blood and cell culture supernatant. Its RNA genome encodes three proteins: the ORF1 replicase, the ORF2 capsid protein and the ORF3 protein involved in virion egress [6]. Previously, we demonstrated that, during the HEV lifecycle, HEV produces several forms of the ORF2 capsid protein [7]: (i) the infectious ORF2i form (also named ORF2c [8]) is the structural component of infectious particles that are likely derived from the assembly of the intracellular ORF2i form, (ii) the glycosylated ORF2g form (also named ORF2s [8]) that is not associated with infectious material but secreted in large amounts (*i.e.,* about 1000 × more than ORF2i [8]) and is the most abundant antigen detected in patient sera [7] and in plasma of HEV-infected human liver chimeric mice [9], and (iii) the ORF2c form that is a cleaved form of ORF2g found in some patient sera and cell culture supernatant [7]. ORF2g and ORF2c (herein referred to as ORF2g/c) likely act as humoral decoys that inhibit antibody-mediated neutralization [8]. Recently, we demonstrated that a 5 amino acid arginine-rich motif (ARM) located in the ORF2 N-terminal region is a unique central regulator of ORF2 addressing that finely controls the HEV lifecycle [10]. Indeed, the ARM controls ORF2 nuclear translocation, promoting regulation of host antiviral responses. This motif also regulates the dual topology and functionality of ORF2 signal peptide, leading to the production of either cytosolic infectious ORF2i or reticular non-infectious glycosylated ORF2 forms. Furthermore, the ARM likely serves as a cleavage site of the glycosylated ORF2 protein. Finally, it promotes ORF2 membrane association that is likely essential for particle assembly [10].

Recent breakthroughs have been achieved in developing cell culture models for HEV [11]. However, many gaps remain in the knowledge of the HEV lifecycle such as the intracellular location of HEV replication and particle assembly as well as the underlying mechanisms of these processes [12]. It is known that the majority of positive sense single-stranded RNA viruses induce host cell membrane rearrangements to facilitate their viral genome replication and viral particle assembly and to protect from the innate immune response. These membrane rearrangements have been well characterized by confocal and electron microscopy approaches leading to the identification of a broad spectrum of complexity between the host membrane remodeling and viral and cellular actors involved in these arrangements [13]. However, due to a scarcity of robust cell culture models and tools, membrane remodeling and viral factories induced by HEV have not been documented yet. Here, we generated monoclonal antibodies directed against the ORF2 capsid protein and used them to probe infectious particles and viral factories in HEV-producing/infected cells.

## Materials and methods

### Generation of monoclonal anti-ORF2 antibodies

Mice immunization and generation of P1H1, P2H1, P2H2 and P3H2 monoclonal antibodies were performed as described in [14]. Briefly, peptides P1 (GQPSGRRRGRRSGG), P2 (AGYPYNYNTTASDQ) and P3 (SRVVIQDYDNQHEQDR) were synthetized and coupled to the protein carrier KLH via a maleimide function by adding a cysteine at the C-terminal position. For the immunization of mice, BIOTEM animal experiments were performed in accordance with the dedicated laws. Institutional Animal Care Committee (IACUC) was DDPP de l’Isère. BIOTEM Ethics committee was the approving committee. Peptides in complete Freund’s adjuvant were injected (20 μg) subcutaneously in OF1 mice. Mice were subsequently immunized twice with peptides (40 μg) in incomplete Freund’s adjuvant. Peptides were administrated intraperitoneally in mice 3 days before cell fusion between splenocytes and myeloma cell line (NS-1). The selection of secreting hybridomas was performed in hypoxanthine–aminopterin–thymidine medium. Before being euthanized using the carbon dioxide method, mice were bled to collect immune serum. Screening of sera, hybridomas and subclones was performed by western-blotting and immunofluorescence on PLC3/HEV cells.

### Antibodies

Primary antibodies used in this study are listed in Table 1. Peroxidase- and fluorochrome-conjugated secondary antibodies were from Jackson ImmunoResearch. Gold-conjugated secondary antibodies were from Aurion (Wageningen, The Netherlands).

**Table 1:** Primary antibodies used in Western blot (WB), immunofluorescence (IF) and immunogold (IG) experiments

Name	Target/Epitope	Host	Isotype	Source	References	Ab Registry	WB	IF	IG
P1H1	ORF2i GQPSGRRRGRRSGG	Mouse	IgG3	This study	n/a	n/a	1/500	1/500	1/100
P2H1	ORF2i AGYPYNYNTTASDQ	Mouse	IgG2a	This study	n/a	n/a	1/500	1/500	1/100
P2H2	ORF2i AGYPYNYNTTASDQ	Mouse	IgG1	This study	n/a	n/a	1/500	1/500	1/100
P3H2	ORF2i/g/c SRVVIQDYDNQHEQDR	Mouse	IgG3	This study	n/a	n/a	1/500	1/500	1/100
1E6	ORF2i/g/c GDSRVVIQDYDNQHEQ DRPTPSPA	Mouse	IgG2b	Millipore	MAB8002	AB_827236	1/2000	1/800	1/100
ORF3	ORF3 ANPPDHSAPLGVTRPSA PPLPHVVDLPQLGPRR	Rabbit	pAb	S. Emerson	[15]	n/a	n/a	1/1000	1/100
H52	Hepatitis C Virus E2 glycoprotein	Mouse	IgG1	J. Dubuisson	[16]	n/a	n/a	n/a	n/a
Tub	β-tubulin C-terminal region	Mouse	IgG1	Sigma	T5201	AB_609915	1/1000	1/100	n/a
MTOC	γ-tubulin N-terminal region	Mouse	IgG1	Sigma	T5326	AB_532292	n/a	1/500	n/a
Calnexin	Human Calnexin	Rabbit		Abcam	ab22595	AB_2069006	n/a	1/1000	n/a
ERGIC53	ERGIC 53 kDa protein	Mouse	IgG1	Enzo Life Sciences Inc	ALX-804-602-C100	AB_2051363	n/a	1/100	n/a
EEA1	Early Endosome Antigen 1	Mouse	IgG1	Transduction Laboratories	610457	AB_397830	n/a	1/500	n/a
Rab5	Rab5	Rabbit	(mAb)	Cell signaling	#3547	AB_2300649	1/1000	1/200	n/a
Rab9a	Rab9a	Rabbit	(mAb)	Cell signaling	#5118	AB_10621426	n/a	1/50	n/a
Rab11	Rab11	Rabbit	(mAb	Cell signaling	#5589	AB_10693925	n/a	1/50	n/a
Rab11a	Rab11a	Rabbit	(pAb)	ThermoFisher	71-5300	AB_2533987	n/a	n/a	1/10
Rab11a	Rab11a	Rabbit	(mAb)	Abcam	ab128913	AB_11140633	1/20000	n/a	n/a
Rab11b	Rab11b	Rabbit	(mAb)	Abcam	ab175925	n/a	1/10000	n/a	n/a
5A6	Tetraspanin CD81 Large extracellular loop	Mouse	IgG1	S. Levy	[17]	AB_627192	n/a	1/300	1/30
CD63	Tetraspanin CD63 Large extracellular loop	Mouse	IgG1	BD Pharmingen	556019	AB_396297	n/a	1/500	n/a
CD71	Transferrin receptor	Mouse	IgG1	Santa Cruz Biotechnology	sc-65882	AB_1120670	n/a	1/100	n/a
CD71	Transferrin receptor	Rabbit	pAb	Abcam	ab84036	AB_10673794	n/a	1/1000	1/100
EHD1	Eps15 homology domain protein 1	Rabbit	(mAb)	Abcam	ab109747	AB_10864800	n/a	1/1000	n/a
MICAL-L1	Molecule Interacting with CasL-like1	Rabbit	IgG	Abcam	ab220648	n/a	n/a	1/100	n/a
PACSIN2	Protein Kinase C and Casein Kinase Substrate in Neurons 2	Rabbit	IgG	MyBiosource	MBS7114698	n/a	n/a	1/1000	n/a
PMP70	70-kDa Peroxisomal Membrane Protein	Mouse	IgG1	Sigma	SAB4200181	AB_10639362	n/a	1/1000	n/a
Catalase	Catalase	Rabbit	IgG	Cell signaling	#12980	AB_2798079	n/a	1/800	n/a
TOM-20	Translocase of the outer mitochondrial Membrane	Mouse	IgG1	BD Biosciences	612278	AB_399595	n/a	1/100	n/a
LAMP1	Lysosomal associated membrane protein 1	Rabbit	(mAb)	Cell signaling	#9091	AB_2687579	n/a	1/200	n/a
V5	GKPIPNPLLGLDST	Mouse	IgG2a	Abcam	ab27671	AB_471093	n/a	1/500	n/a

Graff et al. [15], Flint et al. [16], Oren et al. [17]

### Cells

PLC3 cells are a subclone of PLC/PRF/5 hepatoma cells [7]. PLC3 and Huh-7.5 [18] cells were authenticated by STR profiling and Multiplex Cell Authentication (Multiplexion), respectively, and cultured as previously described [19].

### Plasmids and transfection

The plasmid pBlueScript SK(+) carrying the DNA of the full-length genome of gt3 Kernow C-1 p6 strain (GenBank accession number JQ679013, kindly provided by S.U Emerson) was used [20]. The ORF3-null mutant (HEV-p6-$\Delta$ORF3) and the 5R/5A mutant (HEV-p6-5R/5A) of HEV-p6 were generated as described in [15] and [10], respectively. The plasmid pBlueScript SK(+)/HEV-p6 expressing a V5-tagged ORF1 (V1 insertion) has been described previously [21].

The plasmid pBlueScript SK(+) carrying the DNA of the p6-V1-Puro replicon was generated from the pBlueScript SK(+)/p6-V1 plasmid [21] in which the ORF3/ORF2 region was replaced by a fragment encoding the resistance gene to puromycin as well as the last 321 amino acid residues of ORF2.

Capped genomic HEV RNAs were prepared with the mMESSAGE mMACHINE kit (Ambion) and delivered to PLC3 or Huh-7.5 cells by electroporation using a Gene Pulser Xcell apparatus (Bio-Rad) [7]. PLC3/HEV and Huh-7.5/HEV cells were electroporated with p6 RNA (20 μg/4.10^6^ cells), whereas PLC3 mock and Huh-7.5 mock were electroporated in the absence of RNA.

### Western blotting analyses

Samples were separated by 10% SDS-PAGE and transferred onto nitrocellulose membranes (Hybond-ECL, Amersham). The targeted proteins were detected with specific antibodies (Table 1) and corresponding peroxidase-conjugated secondary antibodies. The detection of proteins was done by chemiluminescence analysis (ECL, Amersham).

### Immunoprecipitations (IP)

Antibodies were bound to Tosylactivated M-280 Dynabead (Thermo Fisher) overnight at 37 °C following the manufacturer’s recommendations. Beads were washed and then incubated for 1 h at room temperature with supernatants (heat-inactivated or Triton-X100-treated), lysates or Triton-X100-treated patient sera. Beads were washed and then heated at 80 °C for 20 min in Laemmli buffer. ORF2 proteins were detected by WB using the 1E6 antibody (Table 1).

### RT-qPCR and qIP

RNAs were extracted and next converted to cDNA by using a polydT primer and the AffinityScript Multiple Temperature cDNA Synthesis kit (Agilent Technologies). qPCR (TaqMan Gene Expression Assay, MGB-FAM-dye, ThermoFisher Scientific) was performed by using the QuantStudio3 Thermocycler (Applied Biosystems) and using primers (5ʹ-GGTGGTTTCTGGGGTGAC-3ʹ (F) and 5ʹ-AGGGGTTGGTTGGATGAA-3ʹ (R)) and a probe (5ʹ-FAM-TGATTCTCAGCCCTTCGC-TAMRA-3ʹ) that target a conserved 70-bp region in the ORF2/3 overlap [22]. For cells harboring the subgenomic p6-replicon, qPCR was performed using primers (5ʹ-AAGACATTCTGCGCTTTGTT-3ʹ (F) and 5ʹ-TGACTCCTCATAAGCATCGC-3ʹ (R)) and a probe (5ʹ-FAM-CCGTGGTTCCGTGCCATTGA-3ʹ) that target the ORF1 [8].

For qIP experiments, samples were first immunoprecipitated as described above. Next, RNAs were extracted and quantified by RT-qPCR as described above.

### Patient samples

Patient samples were collected in France. This was a non-interventional study. Samples were obtained only via standard viral diagnostics following a physician’s order (no supplemental or modified sampling). Data were analyzed anonymously. According to the French law (Loi Jardé), anonymous retrospective studies do not require institutional review board approval.

### Modeling of the N-terminus of ORF2

We used an inhouse ColabFold implementation of AlphaFold2 [23] to generate models of the N-terminus of ORF2. Briefly, we used the ORF2 sequence or only the sequence encompassing up to residue 311 (comprising the shell (S) domain) or up to residue 446 (with the middle (M) domain added), with or without the 13 N-terminal residues (*i.e.* full-length or OR2i), to query the UniRef30 database (March or June 2021 release) with either HHBlits of MMseqs2 [24]. The resulting sequence alignments typically comprised 180–500 hits with 25–400 sequences aligned to any residue of the query. These alignments were used as inputs to AlphaFold2, and 5 models were generated for each. Resulting models were aligned to the S domain of PDB 3iyo. Molecules A, B and C, the three distinct positions in the *T* = 3 icosahedral HEV capsid, were tried. This allowed displaying our models in the context of the capsid-like particle visualized by low-resolution cryo-electron microscopy (cryo-EM, Electron Microscopy Data Bank entry 5173) [25]. The models and EM map were displayed and rendered with the PyMOL Molecular Graphics System (version 1.8 2015).

### PLC3-replicon

Cells stably harboring a p6 subgenomic replicon were generated by electroporating PLC3 cells with p6-V1-Puro replicon-capped RNAs. Cells were next cultured with 2.5 µg/ml of puromycin (Euromedex) for the selection of cells transfected with the ORF1-V5-puro replicon.

### Indirect immunofluorescence

Cells were grown on coverslips in 24-well plates and fixed at 6 d.p.e. for electroporated cells and 12 d.p.i. for infected cells, with 3% of paraformaldehyde (PFA). After 20 min (min), cells were washed twice with phosphate-buffered saline (PBS) and permeabilized for 5 min with cold methanol and then with 0.5% Triton X-100 (TX) for 30 min. Cells were incubated in PBS containing 10% goat serum for 30 min at room temperature (RT) and stained with the indicated primary antibodies for 30 min at RT followed by fluorochrome-conjugated secondary antibodies for 20 min at RT. The nuclei were stained with DAPI (4′,6-diamidino-2-phenylindole). After 2 washes with PBS, coverslips were mounted with Mowiol 4–88 (Calbiochem) on glass slides and analyzed with a LSM 880 confocal laser-scanning microscope (Zeiss) using a Plan Apochromat 63xOil/1.4 N.A. objective. The images were processed using Fiji software.

For high-resolution confocal analyses, images were acquired with an Airyscan module. Z-stacks acquisition were performed with a 0.15-μm z-interval. The confocal parameters were determined to optimize the dynamic range and avoid intensity saturation, and the same settings were applied for each sample. Images in the three channels were recorded using Zen image collection software (Carl Zeiss Microscopy) and processed for high-resolution reconstruction. 3D volumetric surface constructs were obtained using Imaris software (version 9.5.1; Oxford instruments, Belfast), by applying an intensity threshold for each channel. The images were then processed using Fiji software.

### Manders' overlap coefficient (MOC) determination

Colocalization studies were performed by calculating the MOC using the JACoP plugin of Fiji software. For each analysis, at least 30 cells were selected to calculate a MOC mean. A MOC of 1 indicates perfect overlap and 0 no overlap.

### In situ labeling of viral RNA

PLC3 cells electroporated with HEV-p6 (PLC3/HEV) or HEV-p6 expressing a V5-tagged ORF1 (PLC3/HEV-ORF1V5) strains were fixed in 3% PFA for 20 min. Coverslips holding the fixed cells were attached to glass slides with a drop of nail polish, and hydrophobic barriers were drawn around them with the ImmEdge Hydrophobic Barrier Pen (ACD Bio). Next, fixed cells were pre-treated according to the supplier instructions (RNAscope H_2_O_2_ and Protease Reagents). First, the cells were treated with H_2_O_2_ for 10 min at RT and then washed twice with 1PBS. Next, the protease III was diluted 1:15 and incubated for 15 min at RT; slides were washed twice. Then, the RNAscope assay was carried out following the user manual precisely (RNAscope Detection Kit Multiplex Fluorescent Reagent Kit v2 [26, 27]. We used a probe (ref. 1030631-C2, Advanced Cell Diagnostics Bio-Techne) that targets positive strand of genomic viral RNAs [21]. The RNAs were labeled with fluorophore Opal 520 (Akoya Biosciences). Subsequently, immunofluorescent labeling using either P1H1, anti-ORF3 or anti-Rab11 antibodies was performed. Finally, coverslips were mounted and cells were analyzed by confocal microscopy.

### Immunoelectron microscopy

Electroporated cells were fixed by incubation for 2 h with 4% paraformaldehyde in phosphate buffer (pH 7.6), washed twice with PBS (pH 7.6), and centrifuged at 300 × g for 10 min. Cell pellets were embedded in 12% gelatin and infused with 2.3 M sucrose overnight at 4 °C. Ultra-thin cryosections (90 nm) were cut at − 110 °C on a LEICA FC7 cryo-ultramicrotome. The sections were retrieved in a mixture of 2% methylcellulose and 2.3 M sucrose (1:1) and collected onto formvar-/carbon-coated nickel grids. The gelatin was removed by incubation at 37 °C, and the sections were incubated with primary antibodies (Table 1). The grids were washed with PBS and then incubated with secondary antibodies conjugated to gold particles of 6 nm or 10 nm in diameter. The grids were washed with PBS, post-fixed in 1% glutaraldehyde and rinsed with distilled water. Contrast staining was achieved by incubating the grids with a 2% uranyl acetate/2% methylcellulose mixture (1:10). The sections were then examined under a transmission electron microscope operating at 100 keV (JEOL 1011). Electron micrographs were recorded with a digital camera driven by Digital Micrograph software (GMS 3, Gatan).

### Transferrin endocytosis

PLC3/HEV and PLC3/HEV-$\Delta$ORF3 cells were incubated with 25 µg/ml of Alexa633-conjugated transferrin (TrF) at 37 °C and fixed at indicated times. Cells were next permeabilized with methanol and TX and then stained with the P1H1 antibody. Cells were analyzed by confocal microscopy.

### Silencing experiments

At 3 days p.e., PLC3/HEV cells were transfected with small interfering RNA (siRNA) pools (Horizon) targeting Rab11a (ON-TARGETplus human Rab11a, gene 8766, siRNA SMARTpool) and Rab11b (ON-TARGETplus human Rab11b, gene 9230, siRNA SMARTpool) (siRab11) or with a nontargeting siRNA control (siCTL) by using RNAiMax reagent (Invitrogen) according to the manufacturer’s instructions. The knockdown effects were determined at 72 h post-transfection by immunofluorescence, western-blotting, RT-qPCR and virus titration.

### Infectious titers

Huh 7.5 cells were seeded in 96-well plates. The following day, cells were infected with serial dilutions of supernatants or intracellular viral particles from PLC3/HEV cells. Three days post-infection, cells were fixed and processed for indirect immunofluorescence. Cells labeled with anti-ORF2 antibody 1E6 were counted as infected cells. The number of infected cells was determined for each dilution and used to define the infectious titers in focus forming unit/ml. Titers were adjusted to 100% for non-transfected (NT) cells.

### Intracellular viral particles

Confluent T75 flasks of PLC3/HEV cells were trypsinized, and cells were centrifuged for 10 min at 1500 rpm. Cells were washed thrice with PBS. Intracellular viral particles were extracted by resuspending cells in 1 ml of sterile water at room temperature. Cells were vortexed vigorously for 20 min, and then, 110 µl of sterile 10X PBS was added. Samples were clarified by centrifugation 2 min at 14,000 rpm. The supernatants containing intracellular particles were collected and stored at − 80 °C until use.

## Results

### Generation of monoclonal antibodies that specifically recognize the ORF2i protein

**Fig. 1:**
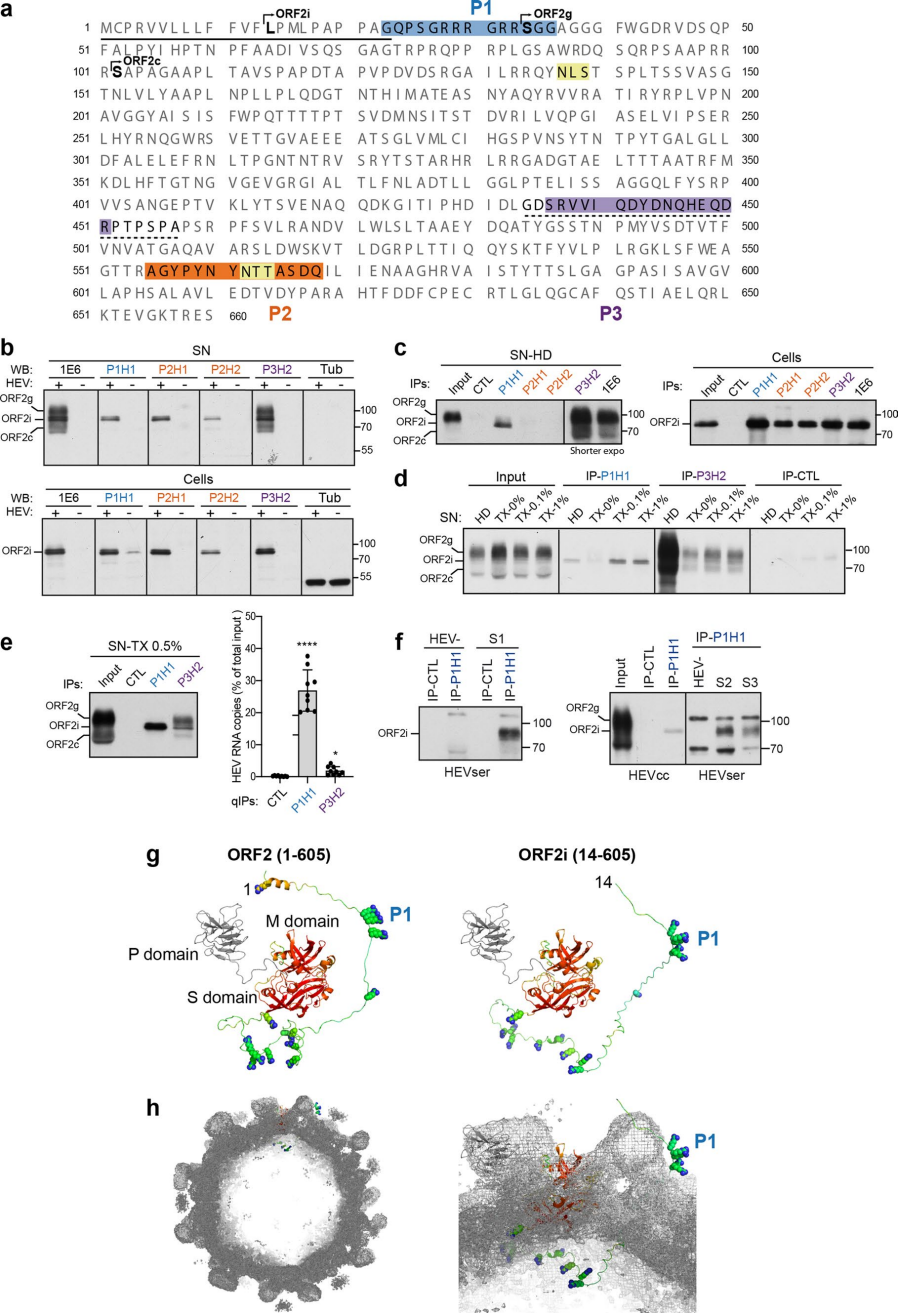
Generation of monoclonal antibodies that specifically recognize the ORF2i protein. **a** Sequence of ORF2 proteins. The line corresponds to the signal peptide. N-glycosylation sites are highlighted in yellow. P1, P2 and P3 peptides are highlighted in blue, orange and purple, respectively. The dashed line corresponds to the 1E6 epitope. **b** Detection of ORF2 proteins in supernatants (SN) and lysates (cells) of PLC3/HEV (+) and mock electroporated PLC3 cells (−) by WB. **c** Immunoprecipitation of ORF2 proteins in heat-denatured (HD, 20 min at 80 °C) SN and lysates of PLC3/HEV cells. An anti-HCV E2 envelope protein antibody (H52) was used as a negative control (CTL). ORF2 proteins were detected by WB with the 1E6 antibody (WB 1E6). **d** IP of SN treated for 30 min with Triton X-100 (TX-0.1%, TX-1%), or left untreated (TX-0%). Inputs used for IP are shown on the left. ORF2 proteins were detected by WB 1E6. **e** SN of PLC3/HEV cells was treated with TX-0.5% and immunoprecipitated with the P1H1, P3H2 or isotype control antibodies. Half of the IP was analyzed by WB 1E6 (left panel) and the other half was processed for RNA extraction and HEV RNA quantification (right panel). Results are expressed as percentage of immunocaptured HEV RNA copies compared to the total input. Values are means from three independent experiments (*n = *3, mean ± S.D., Kruskal–Wallis with Dunn’s test), *p < 0.05, ****p < 0.0001. **f** Sera of HEV-infected (HEVser, S1-S3) or non-infected (HEV-) patients were treated with TX-0.5% and immunoprecipitated with P1H1 or isotype control antibodies. IPs on SN of PLC3/HEV cells (HEVcc) were used as controls. ORF2 proteins were detected by WB 1E6. (b-f) Molecular mass markers are indicated on the right (kDa). For clarity and conciseness concerns, blots were cropped. **g** Models of ORF2 (left) and ORF2i (right) including their N-termini (residues 1–128 and 14–128, respectively. The first residue is labeled. The 606–660 residues located downstream of the P (protruding) domain are not included). Models are displayed in ribbons representation and, for S (shell) and M (middle) domains, colored by expected accuracy from red (most accurate) to blue (least accurate or no defined structure). P (protruding) domain is colored in gray. Arginine side chains upstream residue 128 are displayed as spheres. The P1 epitope is labeled. **h** The same ORF2i model in the same representation and orientation as in (g, right) has been fitted at the 'A' molecule position in the 12-Å cryo-EM map of a virion-sized recombinant ORF2 icosahedral *T* = 3 particle (EMDB 5173). Left, cutaway overall view. Right, zoom on the model

The ORF2 protein sequence contains 660 amino acids (aa) (Fig. 1a). Previously, we demonstrated that the first aa of ORF2i, ORF2g and ORF2c proteins are L^14^, S^34^ and S^102^, respectively [7, 19]. The ORF2i protein is not glycosylated, whereas ORF2g/c proteins are N-glycosylated on ^137^NLS and ^562^NTT sequons [19] (Fig. 1a). We capitalized on these features to design two immunogen peptides (P1 and P2) for obtaining highly specific antibodies of the ORF2i form. The P1 peptide corresponds to the N-terminus of the ORF2i protein that is not present in the ORF2g/c form sequences. The P2 peptide corresponds to 14 aa covering the ^562^NTT sequon that is not occupied by N-glycans on the ORF2i protein, in contrast to ORF2g/c proteins. We also designed a P3 peptide for obtaining antibodies recognizing the different forms of ORF2 (Fig. 1a). To verify the specificity between strains/genotypes, sequence alignments were carried out for each peptide (data not shown). Following mice immunization and hybridoma screening, one clone from P1 immunization (P1H1), two clones from P2 immunization (P2H1 and P2H2) and one clone from P3 immunization (P3H2) were selected and further characterized.

Specificity of generated antibodies was first analyzed in western blotting (WB) experiments with supernatants (SN) and lysates of PLC3 cells electroporated with the gt3 p6 strain (PLC3/HEV cells) and mock electroporated PLC3 cells (Fig. 1b). SN of PLC3/HEV cells contains quasi-enveloped infectious HEV particles (ORF2i) but also large amounts of ORF2g/c proteins, whereas cells express the intracellular ORF2i form that assembles to form intracellular particles [7, 19]. The 1E6 monoclonal antibody, which recognizes the three forms of ORF2 proteins [7], was used as a control. The P1H1, P2H1 and P2H2 antibodies showed a highly specific recognition of the ORF2i protein, which often appears as a doublet, without cross-reacting with the ORF2g/c proteins, as compared to the P3H2 and 1E6 antibodies that recognized the three forms (Fig. 1b).

Antibodies were next used in immunoprecipitation (IP) assays against heat-denatured (HD) SN and lysates of PLC3/HEV cells. In lysates, all antibodies immunoprecipitated the ORF2i protein (Fig. 1c, right panel). In contrast, in SN, no ORF2 proteins were detected in IP-P2H1 and IP-P2H2, whereas the IP-P1H1 showed a highly specific recognition of the ORF2i protein, without cross-reaction with the ORF2g/c proteins (Fig. 1c, left panel), as compared to IP-P3H2 and IP-1E6 that displayed the three forms of ORF2. Thus, P1H1 specifically immunoprecipitates heat-denatured HEV particles.

HEV particles produced in cell culture are wrapped with lipids that mask the ORF2 epitopes. In order to analyze the ability of antibodies to immunoprecipitate non-denatured particles, SN of PLC3/HEV cells was then treated with Triton X-100 (TX), or left untreated (TX-0%) before IP with P1H1 and P3H2 (Fig. 1d). In untreated samples, no ORF2 proteins were detected in IP-P1H1, whereas ORF2g/c proteins were immunoprecipitated by P3H2. In treated samples, the IP-P1H1 showed a highly sensitive and specific recognition of the ORF2i protein, whereas IP-P3H2 displayed the three forms of ORF2. Thereby, P1H1 specifically immunoprecipitates detergent-treated HEV particles.

We next analyzed by RT-qPCR the ability of P1H1 and P3H2 antibodies to immunocapture HEV particles in TX-treated SN. We found that P1H1 immunocaptured 27% of total RNA input, whereas P3H2 immunocaptures only 2% (Fig. 1e). These results indicate that P1H1 efficiently recognizes non-lipid-associated-particles and that its epitope is likely exposed on naked HEV particles.

Importantly, we analyzed the ability of P1H1 antibody to recognize particles from infected patient sera (HEVser). For this purpose, IP-P1H1 and IP-CTL were performed on TX-0.5%-treated sera from HEV-infected (S1-S3) and non-infected (HEV-) patients. As shown in Fig. 1f, P1H1 efficiently immunoprecipitated the particle-associated ORF2i form, indicating that the P1H1 antibody captures patient HEV particles.

Finally, in order to visualize how the P1 epitope may be exposed on HEV particles, we modelled the N-terminal part of ORF2. The N-terminus (residues 1–128 for ORF2 and 14–128 for ORF2i) was clearly flexible and found in different places in different models. However, the very first residues, including P1, were often found on the same level as or even above M and P domains (Fig. 1g), that are exposed at the surface of the icosahedral HEV capsid. Indeed, our models with the most extended N-termini, when placed in the context of the available 12-Å cryo-EM map of the recombinant ORF2 particle [25], readily display the P1 peptide. It is noteworthy that in all models, arginine residues of the N-terminus downstream P1 were still exposed on the inside of the capsid (Fig. 1h).

Antibodies were also tested for their ability to neutralize non-enveloped particles, but none of them displayed neutralizing activity (data not shown), indicating that, while exposed on HEV particles, the P1 epitope is not involved in HEV entry.

### Identification of HEV-induced subcellular structures

**Fig. 2:**
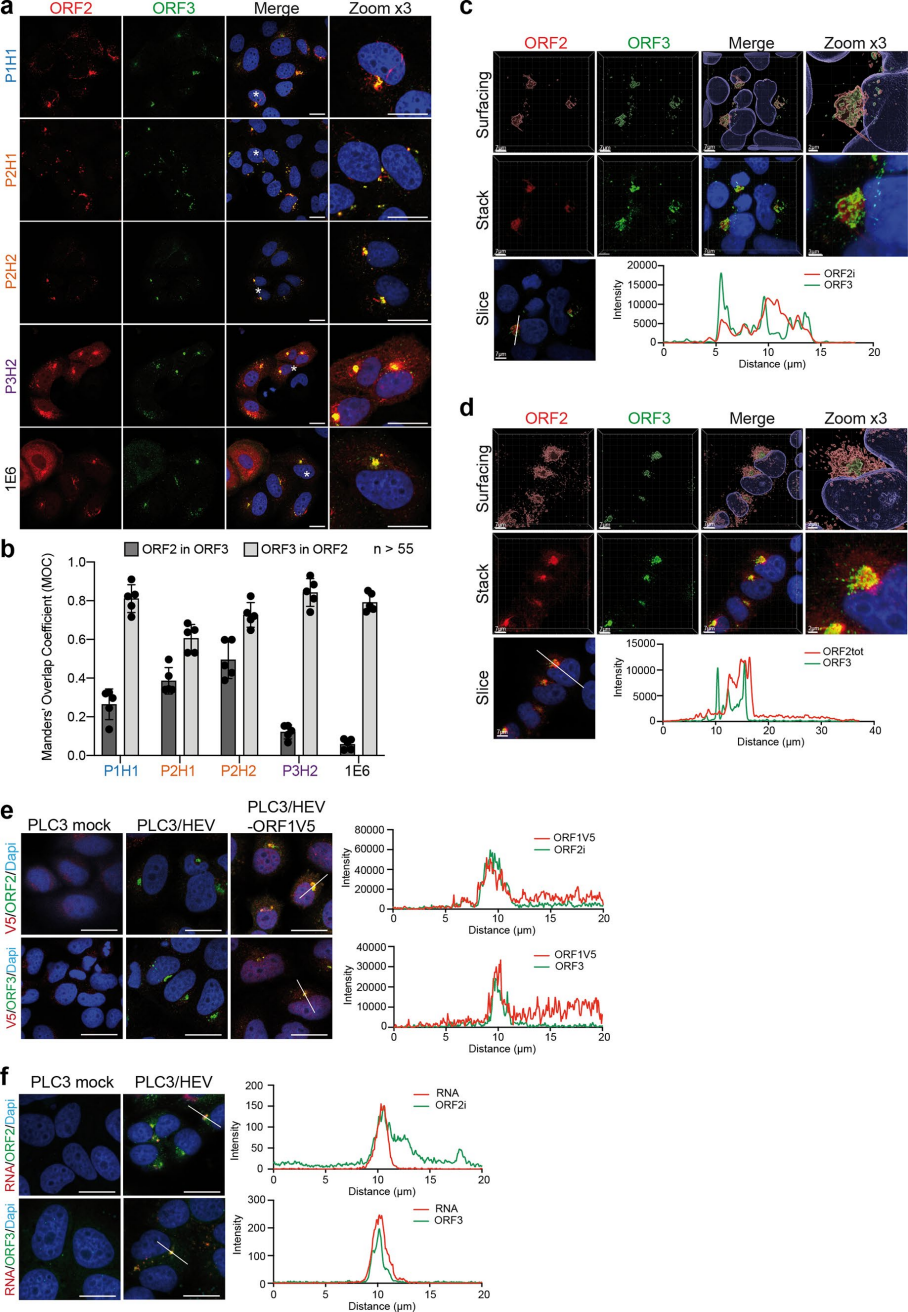
Subcellular structures recognized by anti-ORF2 antibodies. **a** At 6 days post-electroporation (d.p.e.), PLC3/HEV cells were fixed, permeabilized with cold methanol and TX-0.5% and double-stained with indicated anti-ORF2 and anti-ORF3 antibodies. Red = ORF2; Gree*n = *ORF3; Blue = DAPI. Staining were analyzed by confocal microscopy. Scale bar, 20 μm. **b** Manders' overlap coefficients (MOC) of the ORF2 labeling in the ORF3 labeling (ORF2 in ORF3, dark grey) and MOC of the ORF3 labeling in the ORF2 labeling (ORF3 in ORF2, light grey). **c**, **d** PLC3/HEV cells were co-stained with anti-ORF3 and P1H1 (**c**) or P3H2 (**d**) antibodies and analyzed by confocal microscopy with a high-resolution Airyscan module. On the top, volume rendering of the 3D z-stacks (Surfacing) using Imaris is shown to visualize the ORF2/ORF3 substructures. In the middle, z-stacks are shown. On the bottom, line graphs show the fluorescence intensities of ORF2 and ORF3 staining measured every 50 nm across the region of interest highlighted by the white line in the micrograph shown on the bottom left of each panel. Scale bars show the indicated length. **e** PLC3 cells were electroporated with the p6 strain expressing either wildtype (PLC3/HEV) or the V5-tagged ORF1 (PLC3/HEV-ORF1V5) proteins or mock electroporated (PLC3 mock). At 3 d.p.e., cells were processed for immunofluorescence using anti-V5 (V5, red), and P1H1 (ORF2, green) or anti-ORF3 (ORF3, green) antibodies prior to analysis by confocal microscopy. Line graphs show the fluorescence intensities of ORF1V5 and ORF2i or ORF3 staining measured every 70 nm across the region of interest highlighted by the white line in the micrograph shown on the left. **f** PLC3/HEV and PLC3 mock cells were grown on coverslips, fixed at 3 d.p.e. and processed for in situ RNAscope hybridization. Cells were stained with a probe targeting HEV genomic RNA (RNA, red) and P1H1 (ORF2, green) or anti-ORF3 (ORF3, green) antibodies. Line graphs show the fluorescence intensities of RNA and ORF2i or ORF3 staining measured every 70 nm across the region of interest highlighted by the white line in the micrograph shown on the left. Nuclei are in blue. Scale bar, 20 μm

We next performed double-label immunofluorescence and confocal analyses of PLC3/HEV (Fig. 2a) and mock electroporated PLC3 (PLC3 mock) (Fig. S1) cells using the anti-ORF2 antibodies and a polyclonal antibody directed against ORF3, a small protein with a viroporin activity [28] that is associated with the quasi-enveloped viral particle and supports virion egress through the exosomal pathway [29, 30]. ORF3 protein is palmitoylated at cysteine residues in its N-terminal region and is exposed to the cytosolic side of the membrane [31]. The anti-ORF2i antibodies (P1H1, P2H1 and P2H2) mainly displayed a focalized ORF2 staining forming a perinuclear nugget-like structure, whereas the P3H2 and 1E6 antibodies showed a diffuse ER-like ORF2 staining in addition to the perinuclear nugget-like ORF2 staining (Fig. 2a). Of note, P3H2 and 1E6 antibodies recognize the different ORF2 isoforms notably the ORF2g/c forms, which are proteins going through the secretory pathway [7, 10, 19], leading to the diffuse ORF2 staining observed in some cells. Of note, all antibodies also recognized ORF2 proteins from HEV-gt1 (Supplementary data, Fig. S2).

For each antibody, Manders' overlap coefficient (MOC) of either ORF2 staining in ORF3 staining (Fig. 2b, ORF2 in ORF3) or ORF3 staining in ORF2 staining (Fig. 2b, ORF3 in ORF2) was calculated. The ORF3 protein staining highly overlapped with ORF2 for all antibodies, indicating that the ORF3 protein highly colocalizes with the ORF2 proteins (Fig. 2b, MOC ORF3 in ORF2). Moreover, the P1H1, P2H1 and P2H2 antibodies showed a higher MOC of ORF2 in ORF3 than that of P3H2 and 1E6, indicating that ORF3 protein mainly colocalizes with the ORF2i protein (Fig. 2b, ORF2 in ORF3). In line with this, super-resolution confocal microscopy analyses of PLC3/HEV cells stained with either P1H1 (ORF2i, Fig. 2c) or P3H2 (ORF2tot, Fig. 2d) showed a total overlap of fluorescence intensities of ORF2i with ORF3 (Fig. 2c), whereas a shift of fluorescence intensities was observed between total ORF2 and ORF3 proteins (Fig. 2d).

PLC3 cells electroporated with an infectious p6 strain in which a V5 tag was inserted into the ORF1 replicase (PLC3/HEV-ORF1V5, [21]) and double-stained with anti-V5 and P1H1 or anti-ORF3 antibodies (Fig. 2e) also displayed nugget-like structures in which a total overlap of fluorescence intensities of ORF1 with ORF2i (Fig. 2e, upper panel) or ORF1 with ORF3 (Fig. 2e, lower panel) was observed.

Finally, PLC3/HEV and PLC3 mock cells processed for in situ RNAscope hybridization with a probe targeting HEV genomic RNA [21] and stained with P1H1 or anti-ORF3 antibodies (Fig. 2f) demonstrated that viral RNA was also co-distributed with ORF2i (Fig. 2f, upper panel), and ORF3 (Fig. 2f, lower panel) proteins in nugget-like structures in PLC3/HEV cells.

Together, these results indicate that anti-ORF2 antibodies, and more specifically anti-ORF2i antibodies, recognize perinuclear nugget-like ORF2 structures that are in close proximity to ORF3 and ORF1 proteins and viral genome.

**Fig. 3:**
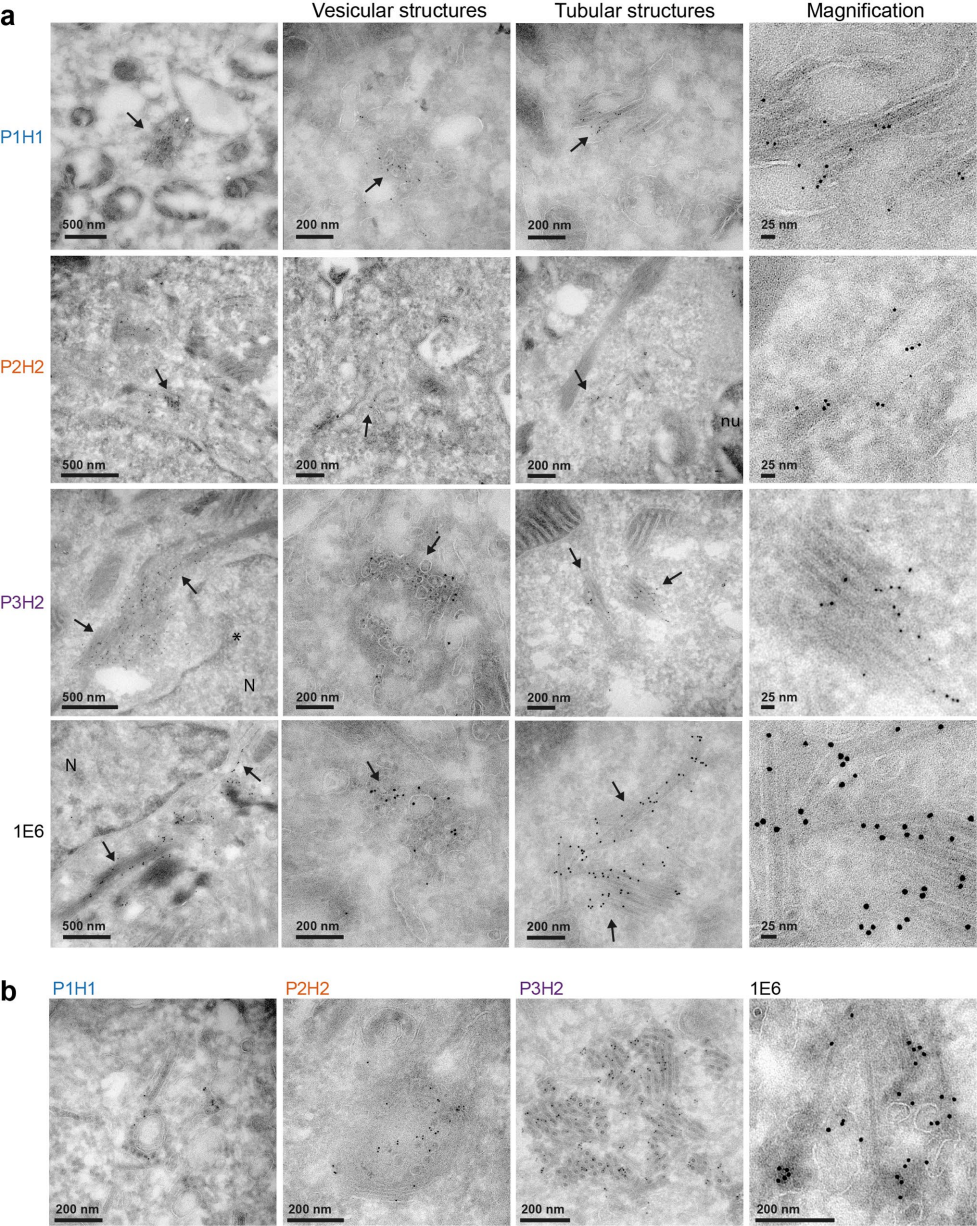
Identification of HEV-induced vesicular and tubular structures in HEV-producing PLC3 cells. **a** Transmission electron microscopy of PLC3/HEV cells cryosections immunogold-labeled with the indicated antibodies. Arrows highlight vesicular and tubular structures. N, nucleus. The asterisk indicates a nuclear deformation. **b** Networks containing both vesicular and tubular structures in PLC3/HEV cells

We then performed electron microscopy (EM) experiments, using immunogold labeling with P1H1, P2H2, P3H2 and 1E6 anti-ORF2 antibodies (visualized by 6-nm gold particles) of ultrathin cryosections of PLC3/HEV (Fig. 3a) and PLC3 mock (Fig. S3) cells. On cryosections of PLC3/HEV cells, the four antibodies specifically labeled two types of subcellular structures, *i.e.,* tubular structures and vesicular structures (Fig. 3a). Tubular structures displayed a homogeneous diameter of 20–25 nm. They were highly organized, often arranged in compact parallel arrays. Vesicular structures were larger and heterogeneous in size with a diameter of 50–250 nm. Both structures were mostly found in the vicinity of the nucleus and led to a nuclear deformation (Fig. 3a, P3H2, asterisk). In most cells, we observed an extensive membrane network containing tubular and vesicular structures (Fig. 3b, P1H1, P2H2 and 1E6), and in some cells, transverse and longitudinal sections of tubular structures were observed (Fig. 3b, P3H2). These results suggest that the perinuclear ORF2-enriched structures previously observed in confocal microscopy likely correspond to the tubular and vesicular structures observed in EM.

**Fig. 4:**
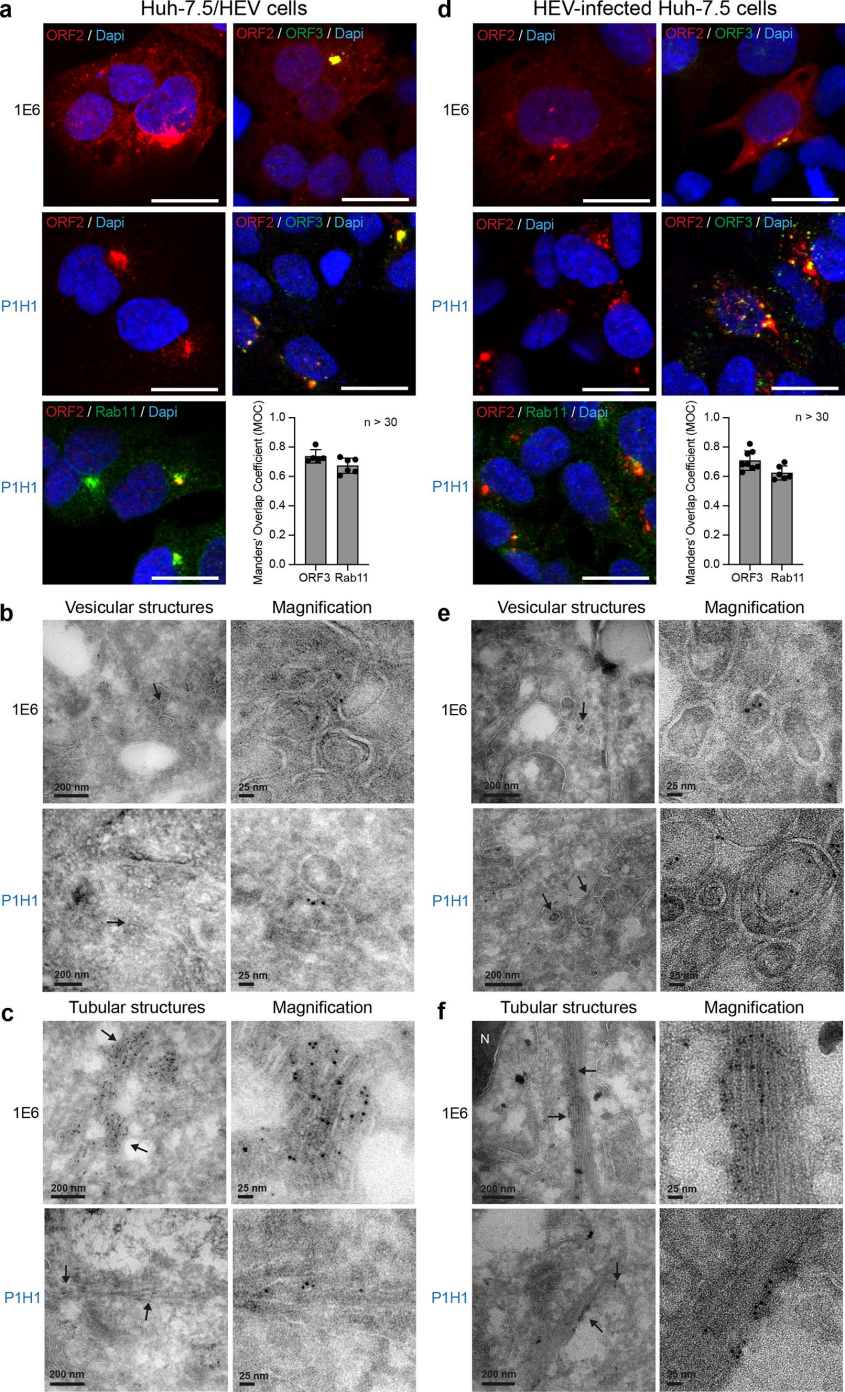
Identification of HEV-induced subcellular structures in HEV-producing Huh-7.5 cells. Huh-7.5 cells electroporated with HEV RNA (**a**–**c**) and Huh-7.5 cells infected with HEV particles (**d**,** f**) were fixed at 6 days p.e and 12 days post-infection, respectively. **a**, **d** Cells were next processed for immunostaining with 1E6, P1H1, anti-ORF3 and anti-Rab11 antibodies, as indicated. Red = ORF2; Green* = *ORF3 or Rab11; Blue = DAPI. Staining were analyzed by confocal microscopy. Scale bar, 20 μm. Manders' overlap coefficients (MOC) of ORF2i (P1H1) staining in ORF3 or Rab11 staining (*n* ≥ 30 cells) were calculated. **b**, **c**, **e–f**) Cryosections of HEV-producing Huh-7.5 cells were processed for immunogold labeling with 1E6 or P1H1 antibodies (visualized by 6-nm gold particles), as indicated. Cryosections were next analyzed by EM. Vesicular (**b**, **e**) and tubular (**c**, **f**) structures containing ORF2 proteins are indicated by black arrows. *N* nucleus

Importantly, confocal analyses of Huh-7.5 cells electroporated with HEV RNA (Fig. 4a) or infected with HEV particles (Fig. 4d) showed that perinuclear nugget-like structures enriched in ORF2i and ORF3 proteins were observed in these cells. In addition, tubular and vesicular structures were identified in EM (Fig. 4b, c and Fig. 4e, f). In contrast, structures were not found in cryosections of mock PLC3 and Huh-7.5 cells (Fig. S3). Together, these results indicate that, during its lifecycle, HEV induces the formation of perinuclear ORF2-enriched ultrastructures in the host cell.

Of note, we have tried to make EM observations under standard fixation and embedding procedures. However, despite numerous observations and in contrast to immunogold labelled cells, we were unable to detect virus-induced ultrastructural changes in the cell sections.

**Fig. 5:**
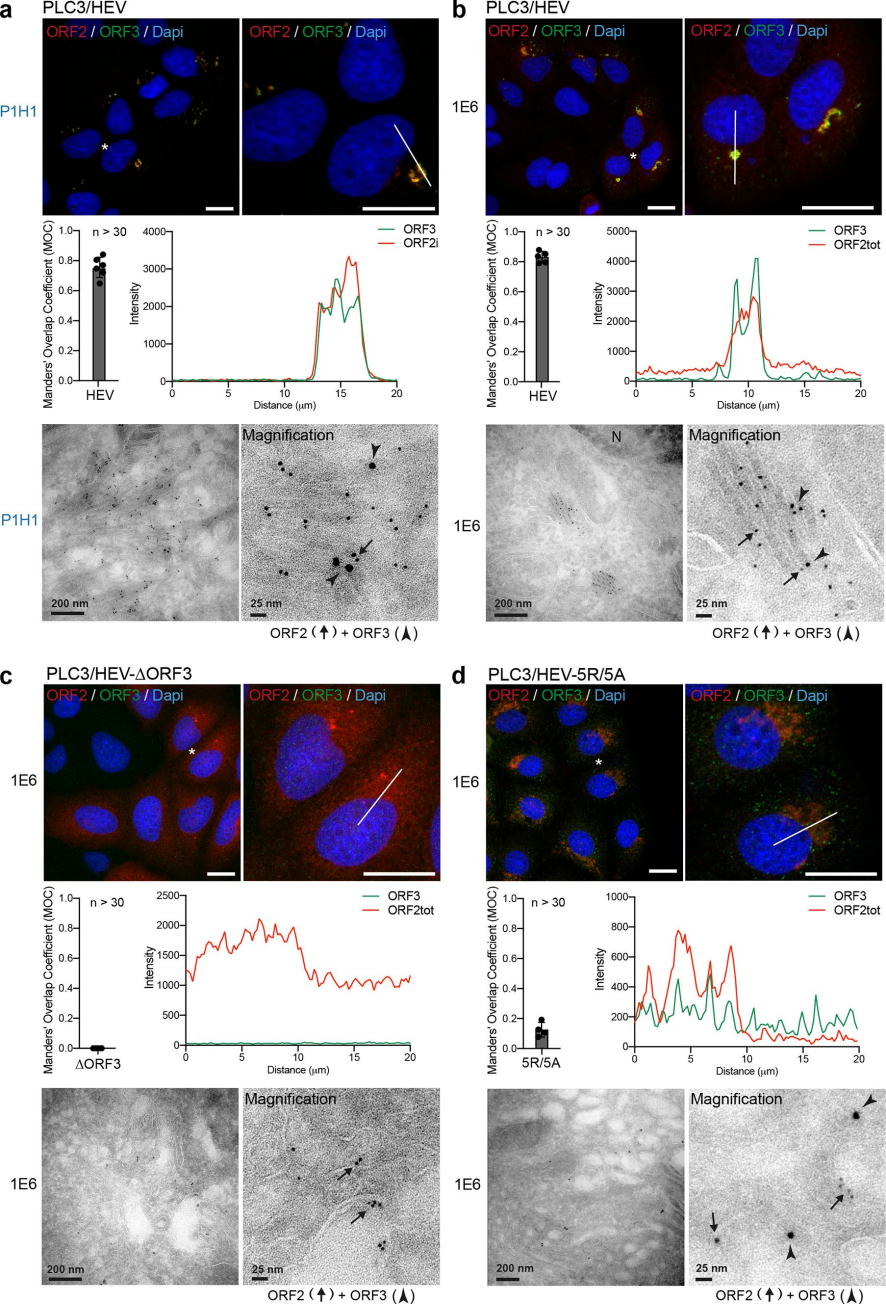
HEV-induced subcellular structures are dependent on the expression of ORF3 protein and assembly of ORF2 capsid proteins. At 6 days post-electroporation (d.p.e.), PLC3/HEV (**a**, **b**), PLC3/HEV-$\Delta$ORF3 (**c**) and PLC3/HEV-5R/5A (**d**) cells were fixed, permeabilized with cold methanol and TX-0.5% and double-stained with P1H1 (**a**) or 1E6 (**b–d**) and anti-ORF3 antibodies. Red = ORF2; Green* = *ORF3; Blue = DAPI. Staining was analyzed by confocal microscopy. Manders' overlap coefficients (MOC) of the ORF3 labeling in the ORF2 labeling were calculated on at least 30 cells. Line graphs show the fluorescence intensities of ORF2 and ORF3 staining measured every 200 nm across the region of interest highlighted by the white line in the micrograph shown above. Scale bar, 20 μm. Cryosections of indicated cells were processed for double immunogold labeling with anti-ORF2 (visualized by 6-nm gold particles) and anti-ORF3 (visualized by 10-nm gold particles) antibodies, as indicated. Cryosections were next analyzed by EM. ORF2 proteins are indicated by black arrows and ORF3 proteins by arrowheads. *N* nucleus. Scale bars show the indicated length

We next performed double immunogold labeling experiments with P1H1 (Fig. 5a) or 1E6 (Fig. 5b) anti-ORF2 (visualized by 6-nm gold particles) and anti-ORF3 (visualized by 10-nm gold particles) antibodies on cryosections of PLC3/HEV cells. We found a co-distribution of ORF3 and ORF2 proteins in vesicular and tubular structures, supporting the confocal microscopy analyses of ORF2/ORF3 co-localization (Fig. 5a, b).

To further understand which viral determinant is important for the formation of HEV-induced subcellular structures, we transfected PLC3 cells with an ORF3-null mutant (HEV-$\Delta$ORF3) [15] (Fig. 5c) and an ORF2 assembly mutant (HEV-5R/5A) [10] (Fig. 5d). In the absence of ORF3 protein expression, HEV particle secretion is abolished [15] and ORF2 proteins mainly accumulate in the cytosol (Fig. 5c). In the HEV-5R/5A mutant, the arginine residues of the ARM located in the P1 epitope (Fig. 1a) were replaced by alanine residues and thus prevent recognition by the P1H1 antibody. The 5R/5A mutations lead to an accumulation of ORF2 in the Golgi apparatus (Fig. 5d) and abrogate viral assembly but do not affect ORF3 expression level [10]. Of note, HEV RNA replication is not altered in PLC3/HEV-$\Delta$ORF3 and PLC3/HEV-5R/5A cells [10].

Strikingly, in the absence of ORF3 expression (PLC3/HEV-$\Delta$ORF3), cells mainly displayed a diffuse ORF2 staining. In EM, immunogold labeling revealed no vesicular or tubular ORF2-enriched ultrastructures but rather ORF2 proteins associated with regular cellular membranes likely derived from ER/GA compartment (Fig. 5c). In PLC3/HEV-5R/5A cells (Fig. 5d), which were characterized by an accumulation of ORF2 in the Golgi and a redistribution of ORF3 protein in the cytosol, we observed no structures resembling the ORF2/ORF3-enriched ultrastructures as those observed in Fig. 5a, b, but both proteins were distributed in the cytosol close to common intracellular vesicles.

Together, these results indicate that, during its lifecycle, HEV induces the formation of subcellular structures that are likely dependent on a fine interplay between ORF2 and ORF3 proteins. Of note, we did not observe any structures resembling the ORF2/ORF3-enriched ultrastructures in PLC3 cells harboring a tagged-ORF1 replicon (data not shown), indicating that these structures are not induced by HEV replication.

### HEV hijacks the endocytic recycling compartment (ERC)

**Fig. 6:**
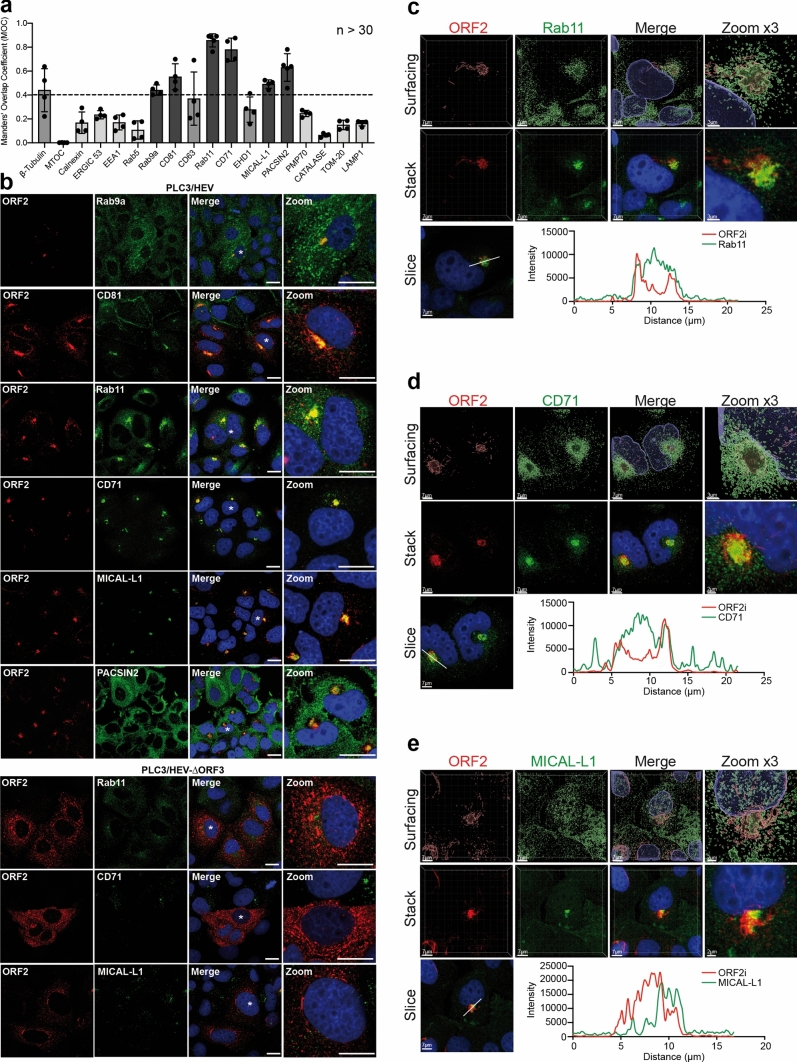
Colocalization analysis of the ORF2i protein with different cell markers. PLC3/HEV and PLC3/HEV-$\Delta$ORF3 cells were fixed, permeabilized with methanol and TX-0.5% and double-stained with P1H1 and anti-cell markers antibodies, as indicated. **a** Manders' overlap coefficients (MOC) of ORF2 and cell marker labeling (*n* > 30 cells). Co-staining showing a low MOC are in light grey and those showing a medium MOC are in middle grey. Co-staining of PLC3/HEV cells showing a MOC > 0.4 are in dark grey and representative confocal images are shown in (**b**, Top). The co-staining of PLC3/HEV-$\Delta$ORF3 cells with P1H1 and antibodies directed against markers of recycling compartment are also shown in (**b**, Bottom). Staining was analyzed by confocal microscopy. Scale bar, 20 μm. PLC3/HEV cells double-stained with P1H1 and anti-Rab11 (**c**), anti-CD71 (**d**) or anti-MICAL-L1 (**e**) antibodies were next analyzed by confocal microscopy with a high-resolution Airyscan module. On the top, volume rendering of the 3D z-stacks (Surfacing) using Imaris is shown to better visualize the stained substructures. In the middle, z-stacks are shown. On the bottom, line graphs show the fluorescence intensities of ORF2i and Rab11/CD71/MICAL-L1 staining measured every 50 nm across the region of interest highlighted by the white line in the micrograph shown on the bottom left of each panel. Scale bars show the indicated length. Red = ORF2; Green = cell marker; Blue = DAPI

To further characterize the HEV-induced subcellular structures, we next carried out an extensive immunofluorescence colocalization study of the ORF2i protein with cell pathway markers. Colocalizations were performed with the P1H1 anti-ORF2i antibody and antibodies directed against markers of cytoskeleton (β-tubulin and MTOC), secretory pathway (Calnexin and ERGIC 53), early endosomes (EEA1 and Rab5), late endosomes / multivesicular bodies (MVB) (Rab9a, CD81 and CD63), Endocytic Recycling Compartment (ERC) (Rab11, CD71, EHD1, MICAL-L1 and PACSIN2), peroxisome (PMP70 and Catalase), mitochondria (TOM-20) and lysosome (LAMP1) (Fig. 6a, b and Fig. S4). Colocalizations were quantitatively analyzed by calculating the MOC (Fig. 6a). As shown in Fig. 6a, b, the ORF2i protein significantly co-distributes with markers of two different cell compartments, i.e., the late endosomes and the ERC. Indeed, the MOCs of ORF2i with Rab9a, CD81 and CD63 were 0.44, 0.55 and 0.37, respectively, indicating a medium colocalization between ORF2i and these cellular markers. Rab9a belongs to a class of small Rab GTPases which are effector proteins promoting exchanges between the late endosome pathway and the trans Golgi network (TGN) [32, 33], whereas CD81 and CD63 are tetraspanins found in MVB, which are a compartment belonging to the late endosome pathway.

**Fig. 7:**
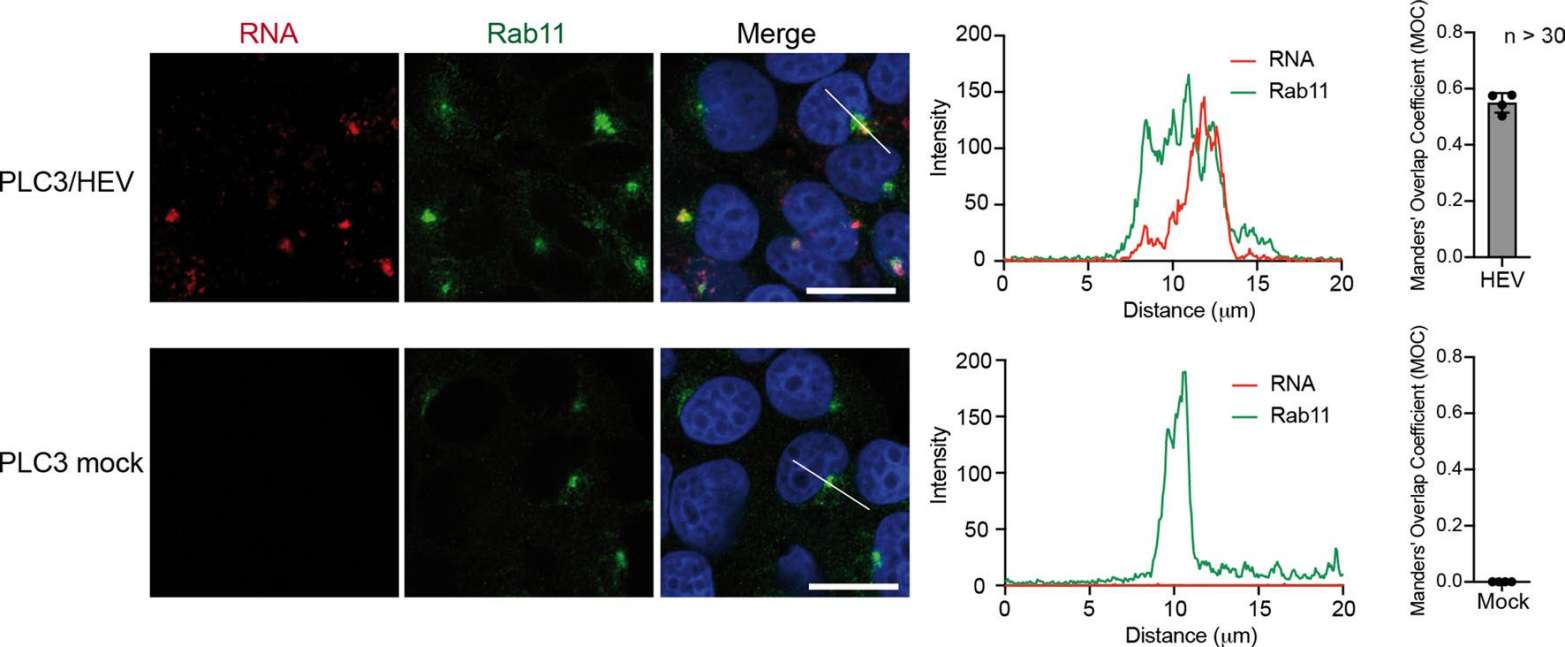
Co-distribution of HEV RNA and Rab11 in PLC3/HEV cells. PLC3/HEV and PLC3 mock cells were grown on coverslips, fixed at 3 d.p.e. and processed for in situ RNAscope hybridization. Cells were stained with a probe targeting HEV genomic RNA (RNA, red) and anti-Rab11 antibody (Rab11, green). Line graphs show the fluorescence intensities of RNA and Rab11 staining measured every 70 nm across the region of interest highlighted by the white line in the micrograph shown on the left. Nuclei are in blue. Scale bar, 20 μm. Manders' overlap coefficient (MOC) of the RNA labeling in the Rab11 labeling was calculated on at least 30 cells

On the other hand, ORF2i strongly colocalized with cell markers of the ERC. This compartment is the keystone of the slow cellular recycling pathway. The ERC plays major roles in cellular metabolism and is subverted during infection by many pathogens such as viruses [34, 35]. The ERC constitutes a collection of tubular organelles that are close to the nucleus and is defined by the presence of the Rab11 GTPase and its effectors. Rab11 regulates recycling from the ERC and transport of cargo from the TGN to the plasma membrane [36–39]. Strikingly, ORF2i and Rab11 showed a MOC of 0.86 (Fig. 6a), indicating a strong colocalization. Indeed, super-resolution confocal microscopy analyses showed a total overlap of fluorescence intensities between ORF2i and Rab11 (Fig. 6c). Moreover, detection of HEV RNA and Rab11 in PLC3/HEV cells demonstrated that viral RNA and Rab11 co-distribute in nugget-like structures (Fig. 7). Of note, ORF2i and Rab11 were also co-distributed in Huh-7.5 cells electroporated with HEV RNA (Fig. 4a) or infected with HEV particles (Fig. 4d).

ORF2i also strongly colocalized with CD71 (MOC = 0.78) (Fig. 6a), the transferrin receptor that is a reference marker for the ERC [40]. This observation was further confirmed by high-resolution microscopy (Fig. 6d).

Efficient recycling via the ERC relies on the integrity of a complex network of elongated, non-symmetrical endosomes known as tubular-recycling endosomes (TRE). A family of proteins known as the C-terminal Eps15 homology domain (EHD1-4) proteins and EHD-interaction partners such as MICAL-L1 (Molecule Interacting with CasL-like1) and PACSIN2/Syndapin2, are involved in TRE biogenesis and control membrane recycling. Although ORF2i only displayed a weak co-localization with EHD1 (MOC = 0.28), it colocalized with MICAL-L1 (MOC = 0.49) and PACSIN2 (MOC = 0.63) (Fig. 6a). The colocalization of ORF2i with MICAL-L1 was further confirmed by high-resolution microscopy but showed a small shift of fluorescence intensities (Fig. 6e), indicating that they are in close proximity to each other. Altogether, our data suggest that HEV likely subverts effectors of the cellular recycling machinery.

On the other hand, although ORF2i did not colocalize with the MTOC (Fig. 6a), ORF2i-enriched structures were found in close proximity of the organizing center (Fig. S4). Of note, it has been shown that the MTOC and the ERC are two distinct structural entities closely related promoting endosomal trafficking [41].

As shown for Rab11, CD71 and MICAL-L1, colocalization analyses in PLC3/HEV-ΔORF3 cells, indicated that, in the absence of ORF3 expression, ORF2i no longer colocalizes with these cell markers (Fig. 6b). These results are in line with our above-described observations on the importance of the ORF3 protein in the formation of HEV-induced subcellular structures (Fig. 5). Although some cellular markers were enriched in the HEV-induced subcellular structures (i.e. CD81 and Rab11), staining of mock electroporated PLC3 cells (PLC3 mock) (Fig. S5) showed similar subcellular localizations of other cell markers as in PLC3/HEV and PLC3/HEV-ΔORF3 cells (Fig. 6), indicating that HEV infection does not induce a general cell marker redistribution.

**Fig. 8:**
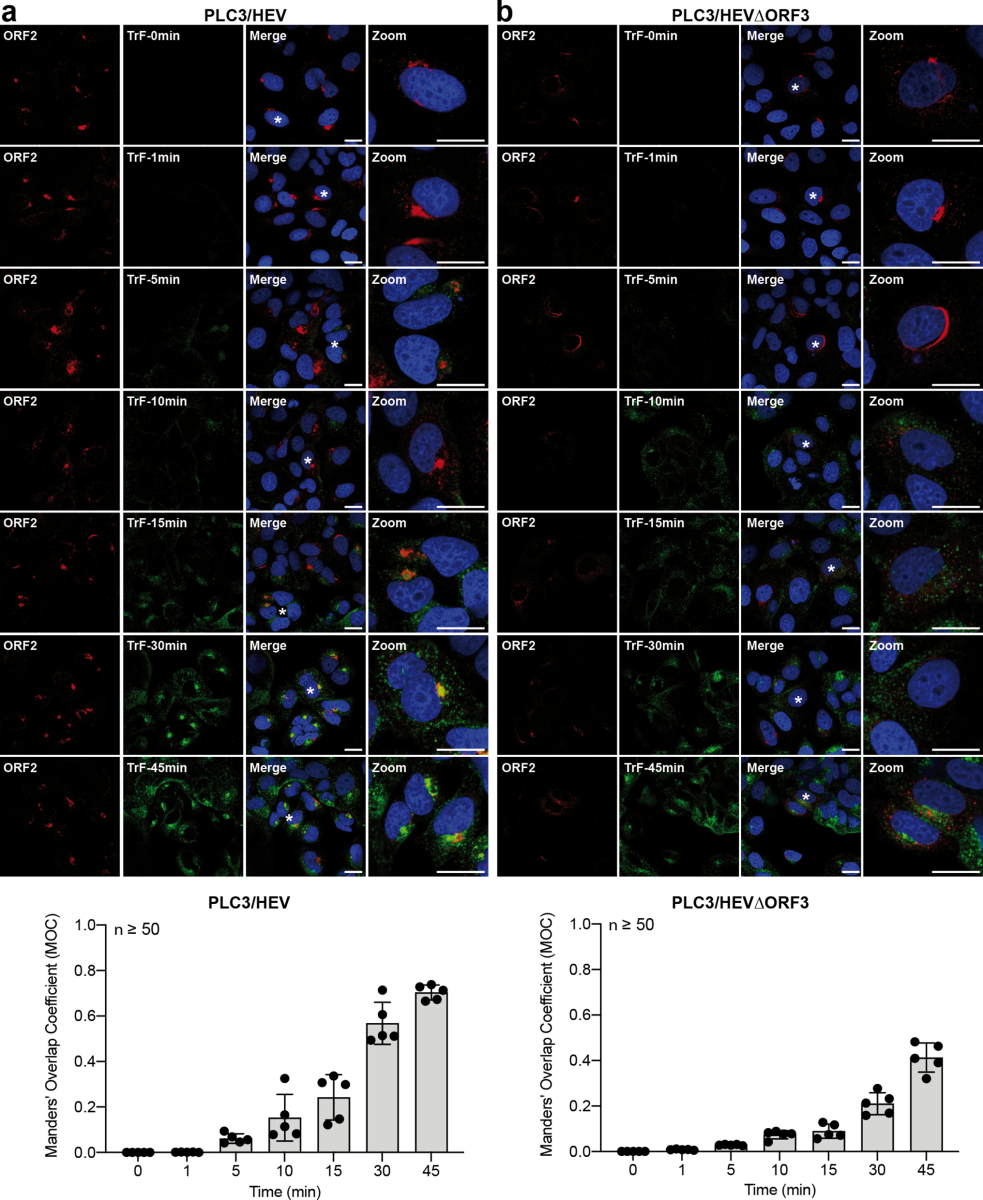
Kinetics of colocalization of the ORF2i protein with transferrin. PLC3/HEV (**a**) and PLC3/HEV-$\Delta$ORF3 (**b**) cells were incubated with fluorochrome-conjugated transferrin (TrF) at 37 °C and fixed at indicated times. Cells were next permeabilized and stained with the P1H1 antibody. Staining was analyzed by confocal microscopy. Red = ORF2; Green = transferrin; Blue = DAPI. Scale bar, 20 μm. Manders' Overlap Coefficients (MOC) of ORF2 staining in TrF staining (*n* ≥ 50 cells) were calculated at each time point

To strengthen our previous observations, we next performed a kinetics of colocalization with the P1H1 anti-ORF2i antibody and fluorochrome-conjugated transferrin in PLC3/HEV and PLC3/HEV-ΔORF3 cells (Fig. 8). It has been shown that Transferrin (TrF) first binds to its receptor (CD71) at the cell membrane and then enters the cell through clathrin-mediated endocytosis. Once in the early endosomes, TrF-CD71 complexes return back to the cell surface through either a fast route going directly back to the plasma membrane, or a slower route delivering TrF-CD71 complexes to the ERC before they are trafficked back to the cell surface [40]. In PLC3/HEV cells, the colocalization between transferrin and ORF2i readily increased over time and reached a MOC of 0.70 after 45 min (Fig. 8a), a value similar to that found in Fig. 6a for its receptor. In contrast, in PLC3/HEV-ΔORF3 cells, transferrin and ORF2i showed a reduced colocalization (MOC = 0.4), (Fig. 8b). Thus, during the HEV lifecycle, the ORF2i protein with the help of ORF3 protein associates with a functional ERC compartment.

**Fig. 9:**
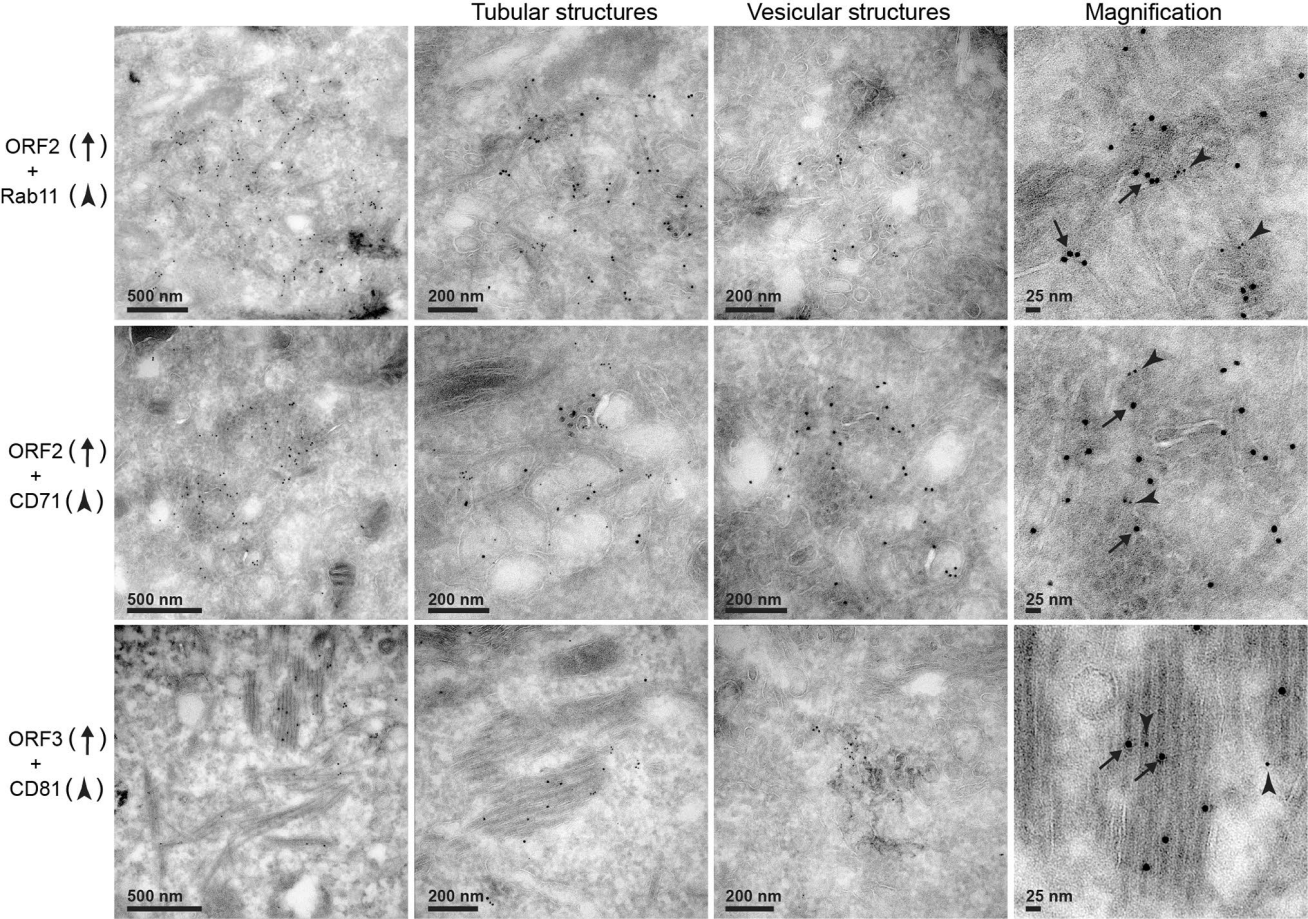
EM analysis of the co-distribution of ORF2 and ORF3 proteins with cell markers. Cryosections of PLC3/HEV cells were processed for double immunogold labeling with 1E6 or anti-ORF3 (visualized by 10-nm gold particles) and anti-Rab11, anti-CD71 or anti-CD81 (visualized by 6 nm gold particles) antibodies, as indicated. Cryosections were next analyzed by EM. ORF2 and ORF3 proteins are indicated by black arrows and cell markers by arrowheads

We carried out double immunogold labeling experiments by combining anti-ORF2 (visualized by 10 nm gold particles) with anti-Rab11 or anti-CD71 (visualized by 6-nm gold particles) antibodies, and anti-ORF3 (10 nm) with anti-CD81 (6 nm) antibodies on cryosections of PLC3/HEV (Fig. 9) and PLC3 mock (Fig. S6) cells. In PLC3/HEV cells, we found a co-distribution of ORF2 with Rab11 and CD71 and a co-distribution of ORF3 with CD81 in vesicular and tubular structures, supporting our confocal microscopy analyses. These results indicate that the HEV-induced vesicular and tubular structures likely derive from ERC and TRE compartments. Moreover, the detection of the transmembrane proteins CD71 and CD81 confirms the presence of membranes in vesicular and tubular structures.

**Fig. 10:**
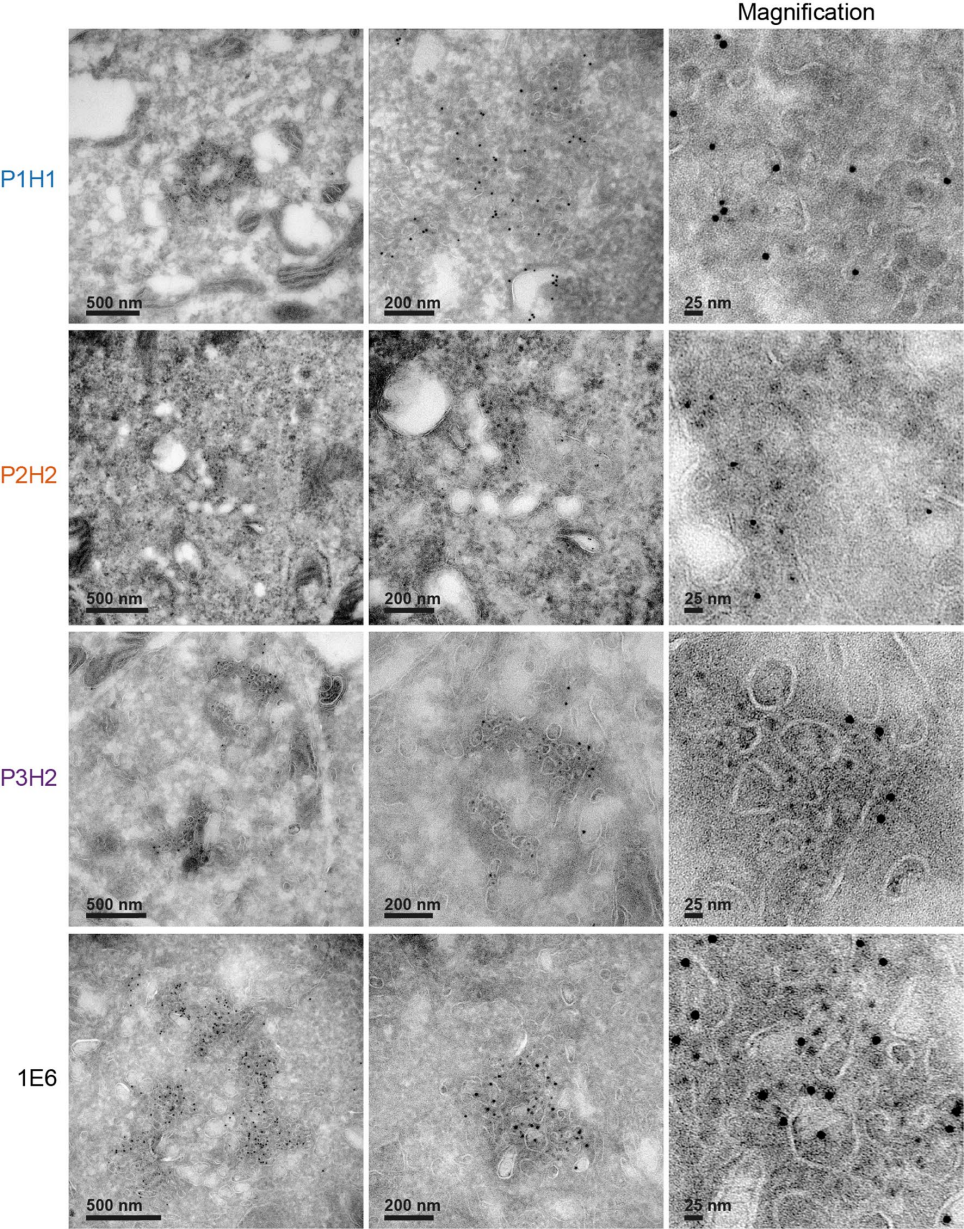
Visualization of intracellular HEV like-particles. Cryosections of PLC3/HEV cells were immunogold-labeled with the indicated antibodies and analyzed by EM

The detection of ORF2i and ORF3 proteins, which are both key players in eHEV biogenesis [7, 10, 42], as well as the ORF1 replicase and HEV RNA in subcellular structures containing Rab11, CD71 and CD81 (a tetraspanin present on the quasi-envelope of eHEV [43]) suggests that the ORF2-enriched ultrastructures we identified likely correspond to eHEV viral factories. In line with this, P1H1, P2H2, P3H2 and 1E6 anti-ORF2 immunolabeling on cryosections of PLC3/HEV cells revealed viral-like particles of ~ 25 nm in diameter in the ORF2-enriched membranous compartments (Fig. 10).

### The ERC plays a central role in the HEV lifecycle

**Fig. 11:**
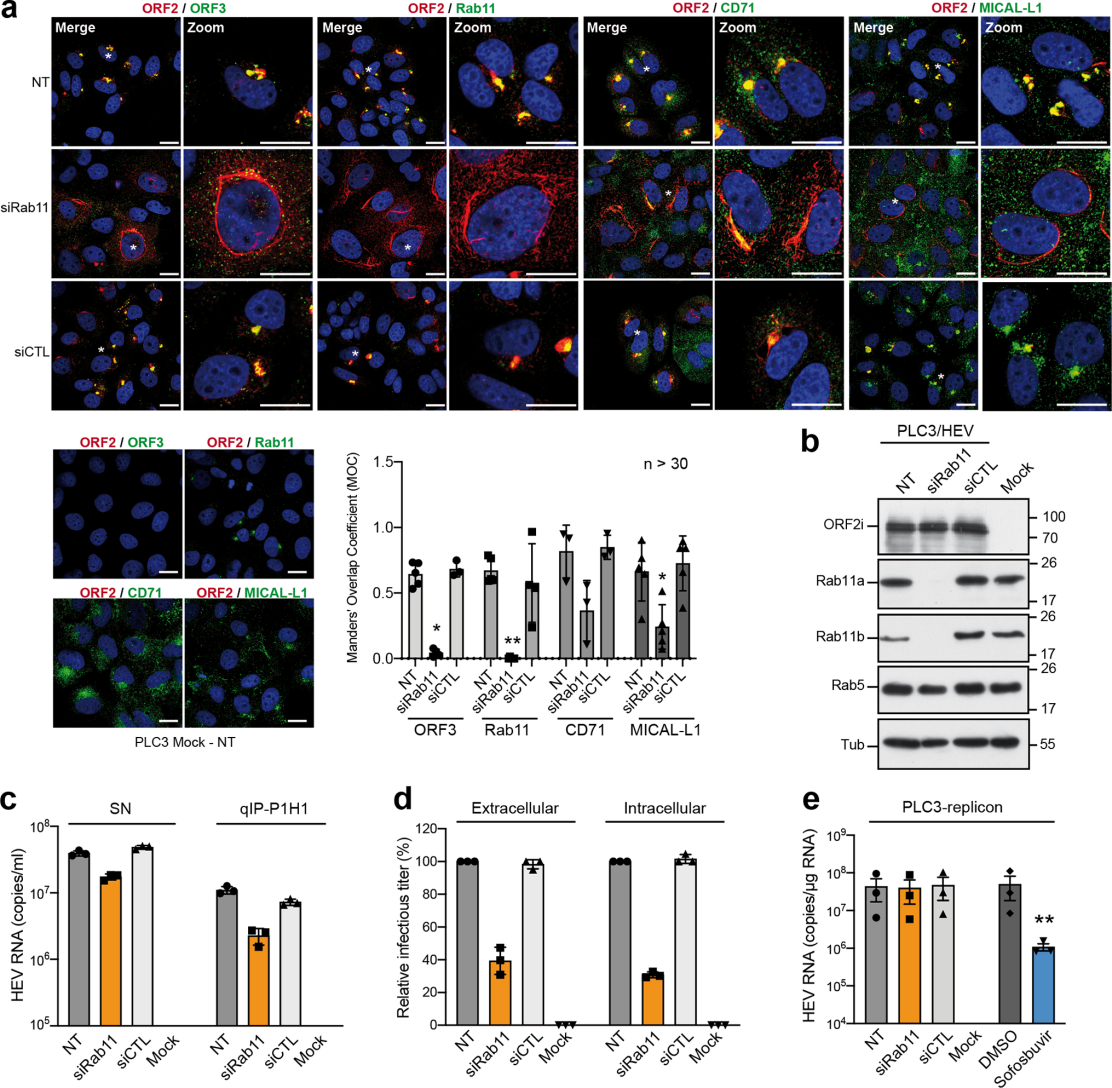
Effect of Rab11 silencing on protein expression and HEV particle secretion. **a**–**d** PLC3/HEV cells were transfected with siRNA targeting Rab11a and Rab11b (siRab11), with a non-targeting siRNA control (siCTL) or left non-transfected (NT). Non-electroporated PLC3 cells were used as controls (Mock). At 72 h post-transfection, cells were analyzed by IF (**a**) and WB (**b**) using the indicated antibodies. Staining were analyzed by confocal microscopy. Scale bar, 20 μm. Red = ORF2i stained by P1H1; Green* = *ORF3 or cell marker; Blue = DAPI. Manders’ overlap coefficients of ORF2i staining in ORF3, Rab11, CD71 or MICAL-L1 staining were calculated. **c** Quantification of HEV RNA in SN of transfected cells was performed by direct RT-qPCR (SN) and after IP with the P1H1 antibody (qIP-P1H1). **d** Production of extracellular and intracellular infectious particles in transfected cells was evaluated by viral titration. **e** PLC3 cells stably replicating a p6 subgenomic replicon were transfected with siRNAs as described above. At 72 h post-transfection, intracellular HEV RNA was quantified by RT-qPCR. Mock cells were used as a negative control. PLC3 cells harboring the replicon and treated with 20 μM of sofosbuvir were used as a positive control for replication inhibition. Values are means from three independent experiments (*n = *3, mean ± S.D., Kruskal–Wallis with Dunn’s test), **p < 0.01

To confirm that the ERC plays a central role in the HEV lifecycle, we conducted functional studies. PLC3/HEV cells were transfected with small interfering RNA (siRNA) targeting Rab11a and Rab11b isoforms (siRab11) or non-targeting siRNA (siCTL) (Fig. 11). The effect of Rab11 silencing on intracellular ORF2 expression was analyzed by IF (Fig. 11a) and WB (Fig. 11b). The effect of Rab11 silencing on viral production was analyzed by quantification of secreted viral RNA (Fig. 11c) and infectious titers (Fig. 11d). The effect of Rab11 silencing on genome replication was analyzed by using PLC3 cells stably harboring a subgenomic replicon (Fig. 11e).

Although Rab11 silencing did not affect the ORF2 expression level, we found that it abrogated the formation of nugget-like structures to the benefit of ORF2-enriched stringy structures mostly localized around the nucleus. These stringy structures did not co-distribute with ORF3, CD71 nor MICAL-L1, which display a more diffuse pattern upon Rab11 silencing (Fig. 11a). Moreover, extracellular RNA levels and infectious titers were reduced in siRab11-transfected PLC3/HEV cells, as compared to control cells (NT and siCTL) (Fig. 11c, d). Although no effect of siRab11 on HEV replication was observed (Fig. 11e), infectious titers showed a significant reduction (i.e., 70%) in intracellular progeny (Fig. 11d), indicating that disruption of the ERC by Rab11 silencing impairs HEV particle assembly. Altogether, these results confirm that the hijacking of the recycling compartment by HEV is pivotal for producing viral particles.

## Discussion

The study performed here with the help of home-made anti-ORF2 antibodies, notably antibodies recognizing the particle associated-ORF2i form, and HEV-producing cells bring new insights into the HEV–host cell interactions.

Previously, we and other demonstrated that during HEV infection, different isoforms of the ORF2 capsid protein are secreted [7, 8]. Indeed, by combining the gt3 p6 strain [20] and the highly transfectable PLC3 cells, we identified 3 forms of the ORF2 capsid protein that perform distinct functions in the HEV lifecycle and display different sequences, post-translational modifications and subcellular localization [7, 19]. The ORF2i form is the component of infectious particles and derives from the assembly of intracellular ORF2i protein [10]. The ORF2i protein is not glycosylated, and its sequence starts at Leu14 corresponding to the middle of the signal peptide (Fig. 1a). Although it has been shown that ORF2i protein might be produced from an additional start codon [8], we recently found that an arginine-rich motif (ARM) located in the ORF2 N-terminal region regulates the dual topology and functionality of ORF2 signal peptide, leading to the production of either the cytosolic infectious ORF2i that is not processed by the signal peptidase or the ORF2g/c forms that are generated by translocation into the ER lumen [10]. The glycosylated ORF2g/c forms, which are not associated with infectious virions and likely act as immunological baits [7, 8], are produced by translocation of ORF2 proteins into the secretory pathway where they are highly glycosylated [19], cleaved by furin [10], and quickly secreted [7, 8]. Here, we capitalized on these distinctive features to generate and characterize four anti-ORF2 monoclonal antibodies, including three antibodies (P1H1, P2H1 and P2H2) directed against the ORF2i form and one antibody (P3H2) directed against the different ORF2 isoforms (Fig. 1). Analyses by WB and IP of HEV-infected cell lysates showed that the four antibodies equally recognize the intracellular ORF2 proteins. In contrast, analyses on HEV-infected cell supernatants, which contain some ORF2i proteins but also huge amounts of ORF2g/c proteins, demonstrated that the P1H1, P2H1 and P2H2 antibodies specifically recognize the ORF2i protein without cross-reaction with the glycosylated ORF2 forms. Importantly, we found that the P1H1 antibody recognizes non-lipid-associated HEV particles from cell culture and patient sera. Therefore, the P1H1 antibody might represent a good candidate for diagnosis purposes. More generally, the antibodies that we generated in this study represent unique tools for deciphering the biogenesis mechanisms of ORF2 isoforms and their precise functions in the HEV lifecycle.

Structure of the ORF2 N-terminal domain has not been resolved yet. It is not included in most of the recombinant constructs that have yielded structural data, and it is disordered in the single structure of full-size particle available [25]. However, based on its potential interaction with RNA and its N-terminal location to the innermost S (shell) domain, this N-terminal domain is thought to be orientated toward the inner cavity of particles. In our study, we found that the P1H1 antibody that recognizes the N-terminal domain of ORF2 (Fig. 1a) efficiently captures delipidated HEV particles (Fig. 1d–f). Although we cannot exclude that the P1H1 antibody recognizes partially assembled ORF2i proteins, our results suggest that the P1 epitope might be exposed on the viral surface. Our structural models showed that this is readily possible (Fig. 1g and h): at least some N-termini may reach as high as or higher than the outer P (protruding) domain and display the P1 epitope, while keeping most other arginine residues directed toward the capsid interior. In addition, we recently found that the ARM located in the ORF2 N-terminal region promotes ORF2 membrane association that is likely essential for particle assembly [10]. Therefore, we hypothesize that the ORF2 N-terminus is associated with eHEV-enveloping lipids and removal of lipids by detergent treatment unmasks ORF2 N-terminal epitopes, including the P1 epitope.

In the present study, we identified by confocal microscopy some HEV-induced subcellular structures that are enriched in viral components. We found that ORF2 and ORF3 proteins highly co-localize in these structures in HEV-producing and HEV-infected cells. Thanks to the P1H1 antibody that specifically recognizes the particle-associated ORF2i protein, we demonstrated that ORF2i and ORF3 proteins are tightly co-distributed in perinuclear nugget-like structures, as well as with the ORF1 replicase and HEV RNA. Interestingly, these structures were not observed in cells harboring a HEV replicon nor in cells expressing only the ORF1 replicase [21], indicating that these structures, although containing ORF1 and viral RNA, are not linked to viral replication. In contrast, we found that in the absence of ORF3 expression, the ORF2i protein was redistributed in cytosolic dot-like structures, and in the absence of ORF2 assembly, the ORF3 protein was also redistributed through the cytosol, suggesting that ORF2i and ORF3 proteins are likely in tight connection and involved in the formation of the perinuclear nugget-like structures.

The ORF2 subcellular localization has been studied in heterologous systems and found in different cell compartments, i.e., plasma membrane, ER and cytoplasmic compartment [44, 45]. Later on, ORF2 characterization in infectious systems led to the identification of the ORF2 isoforms, which are found partitioned in different subcellular compartments. The glycosylated ORF2 forms go through the secretory pathway. The ORF2i form is mainly present in the cytosolic compartment but is also translocated into the nucleus of infected cells [10, 19, 46]. Here, thanks to the specificity of our antibodies, notably the P1H1 antibody, we performed an extensive colocalization study of ORF2i proteins with different cell markers. Of note, as immunofluorescence analysis of ORF2 expression was performed at 6 days p.e., the nuclear localization of the protein could not be shown here as nuclear translocation was reported to occur at early time points p.e. (i.e., 18 h p.e.) [10, 19]. In accordance with previous studies [29, 47], we found a partial co-localization of ORF2i with late endosome markers notably with the tetraspanin CD81, a marker of MVB and exosomes. Importantly, we identified a strong colocalization of ORF2i with several markers of the recycling compartment, including Rab11 and CD71. The subcellular localization of ORF2i was disturbed in the absence of ORF3 expression and upon Rab11 silencing. These results indicate that ORF3 is likely involved in the process of ORF2i addressing to the ERC.

A recent study showed that ORF3 is palmitoylated at cysteine residues in its N-terminal region and is exposed to the cytosolic side of the intracellular membranes [31]. It has also been localized in early and recycling endosomes [48] as well as in MVB [29] and has been found in association with microtubules [49, 50]. Hence, ORF3 may behave as a cargo able to drive ORF2 to HEV factories.

The ORF1 protein is the viral replicase, the ORF2i protein is the structural subunit of HEV virion, and the ORF3 protein plays an essential role in exosomal release and acquisition of the quasi-envelope around the neo-synthesized viral particles. Here, we showed that the 3 viral proteins as well as the genomic RNA were co-distributed in HEV-producing cells in a cellular compartment probably derived from the ERC. Our findings strengthen the hypothesis of a close connection between HEV replication and assembly sites [51]. However, although ORF1 and RNA were detected, the identified structures are not required for HEV replication, as demonstrated by Rab11 silencing. Therefore, we hypothesize that the observed structures represent viral factories in which viral components are in close proximity and assemble into particles which acquire their membrane through an ORF2-ORF3 interaction.

Ultrastructural analyses by EM of cryosections of HEV-replicating cells showed that the ORF2/ORF3-enriched structures correspond to a network of vesicular and tubular components located in the vicinity of the nucleus and in which Rab11, CD71 and CD81 were found. On the one hand, tubular structures formed regular parallel arrays and displayed a homogeneous diameter of 20–25 nm, corresponding to that of intracellular HEV-like particles observed in cryosections. On the other hand, vesicular structures were heterogeneous in their organization and their size, i.e., 50–250 nm, and displayed some viral-like particles. Although we cannot exclude that the tubular structures correspond to a dead-end pathway containing viral and cellular proteins, we speculate that these structures might correspond to virion precursors containing assembled or pre-assembled virions while vesicular structures might correspond to a later compartment of HEV virion assembly. It should be noted that vesicular and tubular structures were found in both PLC3/HEV and Huh-7.5/HEV cells, indicating that they are not cell type specific. Furthermore, we found the same structures in HEV-infected Huh-7.5 cells, indicating that they do not correspond to an artifact of cell electroporation. Although difficult to set up, it would be interesting to analyze these structures in correlative microscopy approaches as well as to find out if this kind of structures form in a heterologous expression system and in other infectious models such as gt1 replicating cells or in HEV-infected primary cells.

ERC is involved in several stages of the lifecycle of a number of DNA and RNA viruses, with Rab11 being a central player in most of these processes [35]. ERC notably mediates viral transport, assembly and egress, e.g., it is involved in the envelopment of herpes simplex-1 capsids [52] or contributes to the transport of HCV virions towards the plasma membrane [53]. Here, we demonstrated that HEV particle assembly depends on a functional ERC. Interestingly, it has been shown that ERC hijacking is associated with membrane remodeling upon infection. Cholesterol accumulates at the ERC during Influenza A virus (IAV) infection, or Rab11 redistributes from dot structures to large aggregates during infection with several viruses including IAV [35]. Ultrastructural changes of these ERC membrane remodeling were poorly investigated to date. In our study, we demonstrated that HEV is a new candidate in the list of viruses hijacking the ERC. Importantly, we found that viral proteins and recycling compartment markers are co-distributed in perinuclear structures found in ultrastructural analyses as a network of vesicular and tubular structures. To our knowledge, this kind of structures has never been described before and might be the hallmark of HEV infection.

## Supplementary Information

Below is the link to the electronic supplementary material.

Supplementary file1 (PDF 5571 kb)

## Data Availability

The datasets generated and analyzed during the current study are available from the corresponding author on reasonable request.

## Abbreviations

aa: Amino acid
ARM: Arginine-rich motif
CTL: Control
EEA1: Early endosome antigen-1
EHD: Eps15 homology domain
eHEV: Enveloped HEV
EM: Electron microscopy
ER: Endoplasmic reticulum
ERC: Endocytic-recycling compartment
ERGIC: Endoplasmic reticulum-Golgi intermediate compartment
ESCRT: Endosomal sorting complexes required for transport
GA: Golgi apparatus
gt: Genotype
HD: Heat-denatured
HEV: Hepatitis E virus
IAV: Influenza A virus
IF: Immunofluorescence
IG: Immunogold
IP: Immunoprecipitation
LAMP1: Lysosomal-associated membrane protein 1
MICAL-L1: Molecule interacting with CasL-like1
MOC: Mander’s overlap coefficient
MTOC: Microtubule-organizing center
MVB: Multivesicular bodies
NT: Non-transfected
ORF: Open-reading frame
ORF2c: Cleaved ORF2
ORF2g: Glycosylated ORF2
ORF2i: Infectious ORF2
PACSIN2: Protein Kinase C and Casein Kinase Substrate in Neurons 2
p.e.: Post-electroporation
PMP70: 70-KDa peroxisomal membrane protein
qIP: Immunoprecipitation followed by RT-qPCR
siRNA: Small-interfering RNA
SN: Supernatant
TGN: Trans Golgi network
TRE: Tubular-recycling endosome
TrF: Transferrin
TX: Triton X-100
WB: Western blotting
