# Supplementary material for: The endocytic recycling compartment serves as a viral factory for hepatitis E virus
Source: Cell Mol Life Sci. 2022 Dec 3;79(12):615. doi: 10.1007/s00018-022-04646-y (PMC9718719; doi:10.1007/s00018-022-04646-y)
Supplement: Supplementary file 1 — Supplementary file1 (PDF 5571 kb) [file 18_2022_4646_MOESM1_ESM.pdf]

Supplementary data

The Endocytic Recycling Compartment Serves as a Viral Factory for Hepatitis E Virus

Cyrine Bentaleb[a,#], Kévin Hervouet[a,#], Claire Montpellier[a,§], Charline Camuzet[a,§], Martin Ferrié[a,§], Julien Burlaud-Gaillard[b,c], Stéphane Bressanelli[d], Karoline Metzger[a], Elisabeth Werkmeister[e], Maliki Ankavay[a,¶], Nancy Leon Janampa[b], Julien Marlet[b], Julien Roux[f], Clarence Deffaud[f], Anne Goffard[a], Yves Rouillé[a], Jean Dubuisson[a], Philippe Roingeard[b,c], Cécile-Marie Aliouat-Denis[a,$] and Laurence Cocquerel[a,$,*]

[a] University of Lille, CNRS, Inserm, CHU Lille, Pasteur Institute of Lille, U1019-UMR 9017-CIIL- Center for Infection and Immunity of Lille, F-59000 Lille, France.

[b] Inserm U1259. Morphogénèse et Antigénicité du VIH et des Virus des Hépatites (MAVIVH), Université de Tours and CHRU de Tours, 37032 Tours, France.

[c] Université de Tours et CHRU de Tours, Plateforme IBiSA de Microscopie Electronique, Tours, France

[d] Université Paris-Saclay, CEA, CNRS, Institute for Integrative Biology of the Cell (I2BC), Gif-sur-Yvette, France.

[e] Univ. Lille, CNRS, Inserm, CHU Lille, Institut Pasteur de Lille, UMR2014 - US41 - PLBS-Plateformes Lilloises de Biologie & Santé, Lille, France.

[f] BIOTEM, Apprieu, France

[#]C.B. and K.H. contributed equally to this work

[§]C.M., C.C. and M.F. contributed equally to this work

[$]C-M.A-D. and L.C. contributed equally to this work

¶Present address: Division of Gastroenterology and Hepatology, Institute of Microbiology, Lausanne, Switzerland

*Corresponding author: Laurence Cocquerel, University of Lille, CNRS, INSERM, CHU Lille, Pasteur Institute of Lille, U1019-UMR 9017-CIIL- Center for Infection and Immunity of Lille, F-59000 Lille, France, laurence.cocquerel@cnrs.fr

Content

Supplementary results

Supplementary Materials and methods
Cells
Plasmids and transfection

Supplementary Figures and legends

Supplementary Fig. 1: ORF2 and ORF3 antibody recognition in PLC3 mock cells.

Supplementary Fig. 2: Antibody recognition of gt1 and gt3 ORF2 proteins.

Supplementary Fig. 3: Immunogold labeling of PLC3 and Huh-7.5 mock cells.

Supplementary Fig. 4: Colocalization analysis of the ORF2i protein with different cell markers in PLC3/HEV cells.

Supplementary Fig. 5: Colocalization analysis of the ORF2i protein with different cell markers in PLC3 mock cells.

Supplementary Fig. 6: Double-immunogold labeling of PLC3 mock cells.

References

Supplementary results

Antibody recognition of gt1 and gt3 ORF2 protein.

Since no efficient cell culture model of HEV gt1 is currently available, we used a heterologous expression system to compare antibody recognition towards gt1 and gt3 ORF2 proteins. Sar55 (gt1) and p6 (gt3) ORF2 sequences were cloned into pTM plasmids and expressed in Huh-7 cells stably expressing the T7 RNA-polymerase (H7-T7-IZ cells) [1]. As shown in **Fig. S2**, although the ORF2-gt3 pattern was different from that observed in infectious system, the ORF2 recognition pattern for each antibody was similar for gt1 and gt3 ORF2-expressing cells. These results indicate that antibodies also recognized ORF2 proteins from HEV-gt1.

Supplementary Materials and methods

Cells. The Huh-7-derived H7-T7-IZ cells stably expressing the T7 RNA polymerase ([1]; kindly provided by Ralf Bartenschlager, University of Heidelberg, Germany) were maintained in a medium supplemented with 50 µg/ml of Zeocin. They were used for the transfection of the T7 promoter-driven pTM expression vectors. H7-T7-IZ cells were authenticated by Multiplex Cell Authentication (Multiplexion).

Plasmids and transfection. The pTM-ORF2-HEV-gt3 plasmid was kindly provided by J. Gouttenoire (University of Lausanne, Switzerland) [2]. The pTM-ORF2-HEV-gt1 plasmid was generated by cloning the Sar55 strain ORF2 sequence into the multiple cloning site of pTM plasmid. Plasmids were transfected into H7-T7-IZ cells using ViaFect Transfection Reagent (Promega) following the manufacturer's recommendations.

Supplementary figures and legends

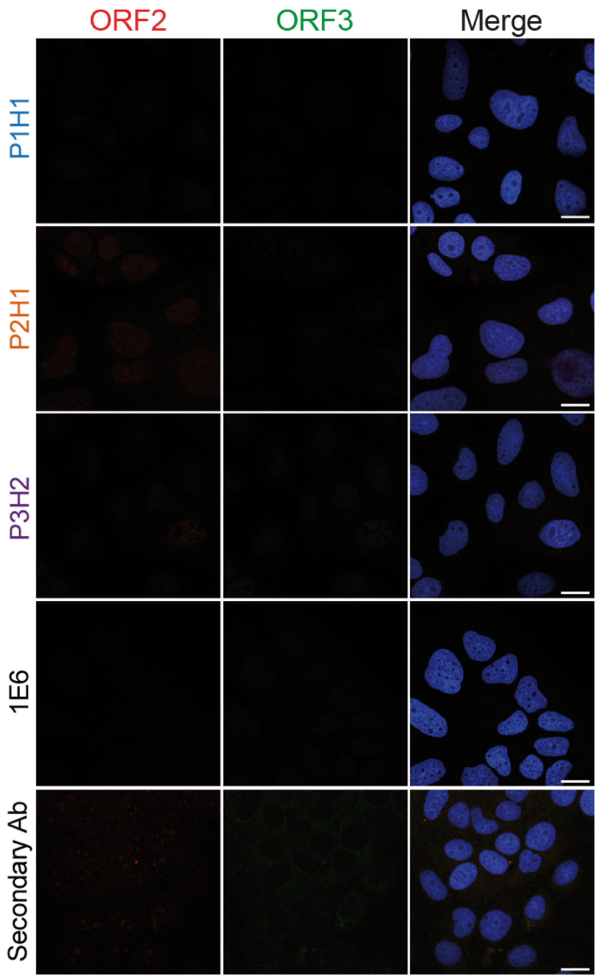

Supplementary Fig. 1: ORF2 and ORF3 antibody recognition in PLC3 mock cells. Mock electroporated PLC3 cells were fixed, permeabilized with methanol and TX-0.5% and double-stained with indicated anti-ORF2 and anti-ORF3 antibodies or with secondary antibodies only. Red = ORF2; Green = ORF3; Blue = DAPI. Staining were analyzed by confocal microscopy. Scale bar, 20μm.

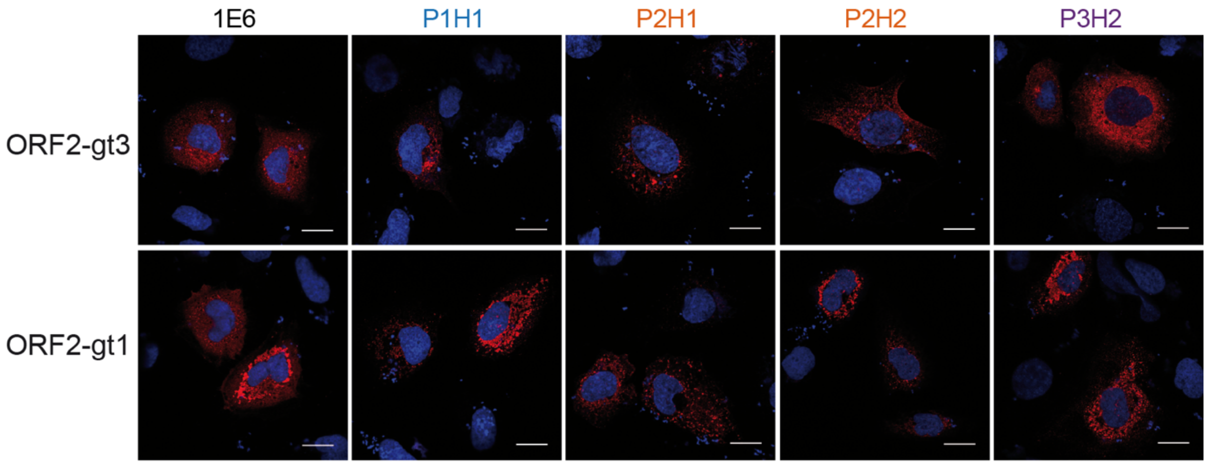

Supplementary Fig. 2: Antibody recognition of gt1 and gt3 ORF2 proteins. H7-T7-IZ cells transfected with pTM plasmid expressing p6 strain ORF2 (ORF2-gt3) or Sar55 strain ORF2 (ORF2-gt1) were fixed at 16h post-transfection, permeabilized with Methanol and TX-0.5% and stained with indicated anti-ORF2 antibodies. Staining were analyzed by confocal microscopy. Red = ORF2; Blue = DAPI. Scale bar, 20μm.

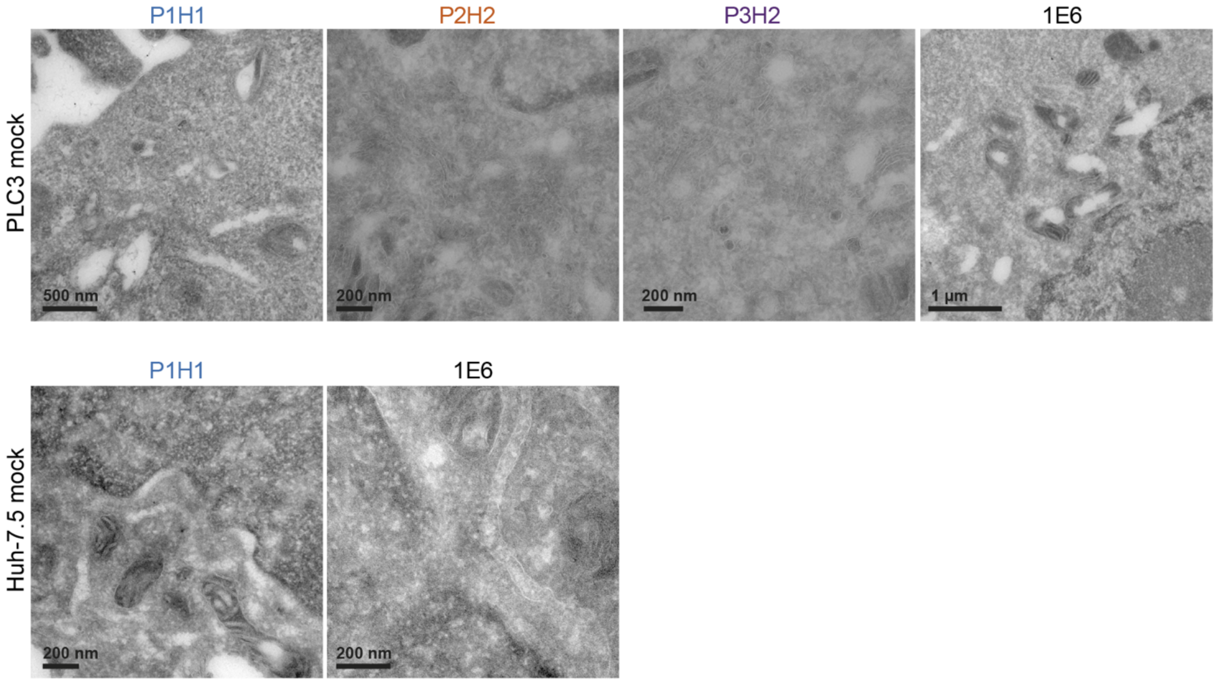

Supplementary Fig. 3: Immunogold labeling of PLC3 and Huh-7.5 mock cells. Cryosections of PLC3 and Huh-7.5 mock cells were immunogold-labeled with the indicated antibodies and analyzed by EM.

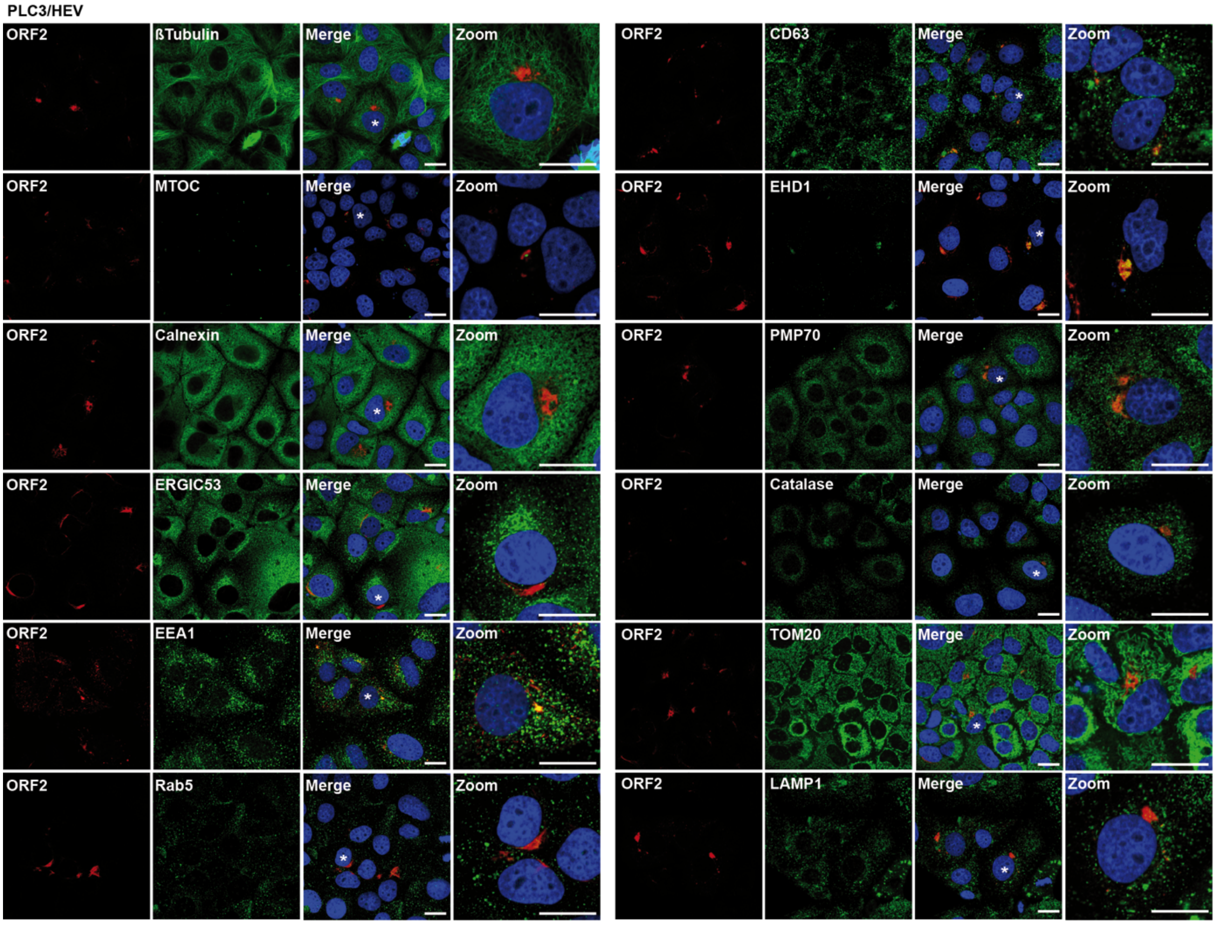

Supplementary Fig. 4: Colocalization analysis of the ORF2i protein with different cell markers in PLC3/HEV cells. PLC3/HEV cells were fixed, permeabilized with cold methanol and TX-0.5% and double-stained with P1H1 and anti-cell marker antibodies, as indicated. Staining were analyzed by confocal microscopy. Scale bar, 20µm.

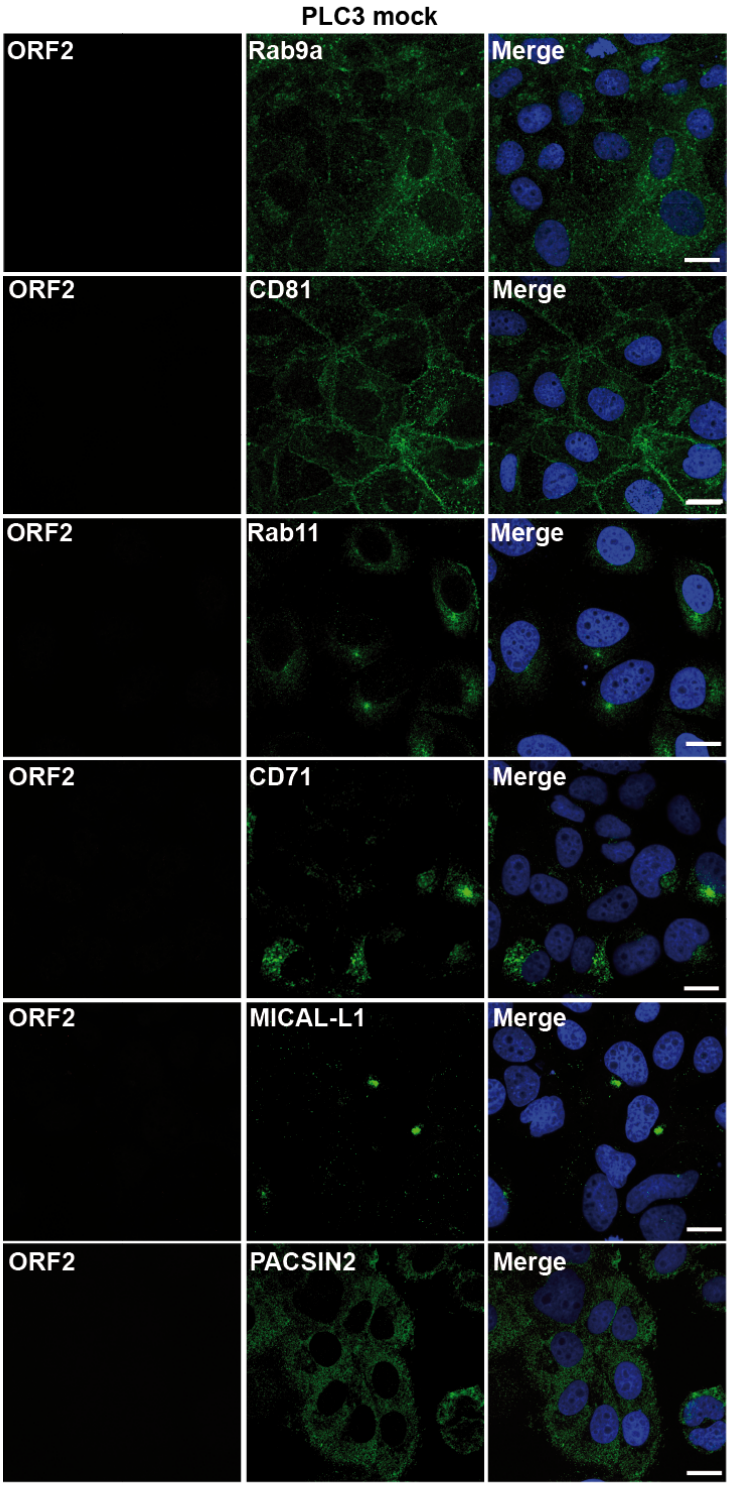

Supplementary Fig. 5: Colocalization analysis of the ORF2i protein with different cell markers in PLC3 mock cells. PLC3 mock cells were fixed, permeabilized with methanol and TX-0.5% and double-stained with P1H1 and anti-cell marker antibodies, as indicated. Staining were analyzed by confocal microscopy. Scale bar, 20µm.

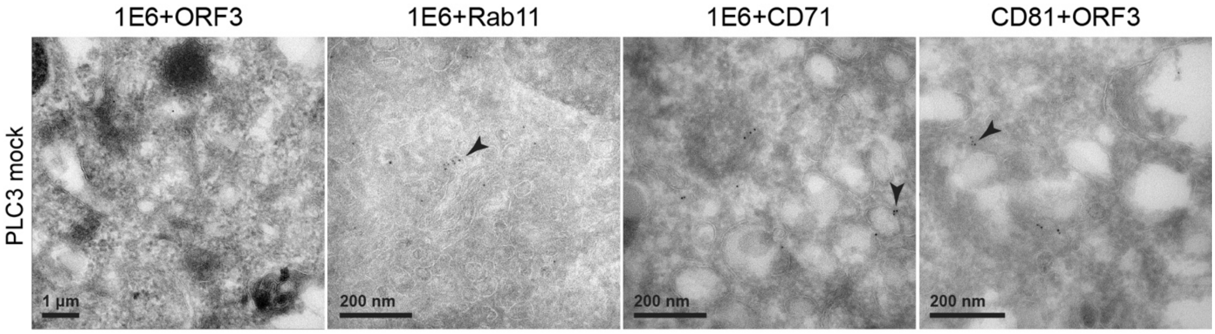

Supplementary Fig. 6: Double-immunogold labeling of PLC3 mock cells. Cryosections of PLC3 mock cells were processed for double immunogold labeling with anti-ORF2 (visualized by 6 nm gold particles) and anti-ORF3 (visualized by 10 nm gold particles) or anti-ORF2 (visualized by 10 nm gold particles) and anti-Rab11 or anti-CD71 (visualized by 6 nm gold particles), anti-ORF3 (visualized by 10 nm gold particles) and anti-CD81 (visualized by 6 nm gold particles) antibodies, as indicated. Cryosections were next analyzed by EM. Arrowheads indicate cell markers.

References

1. Romero-Brey I, Merz A, Chiramel A, et al (2012) Three-dimensional architecture and biogenesis of membrane structures associated with hepatitis C virus replication. PLoS Pathogens 8:e1003056. https://doi.org/10.1371/journal.ppat.1003056

2. Lenggenhager D, Gouttenoire J, Malehmir M, et al (2017) Visualization of hepatitis E virus RNA and proteins in the human liver. Journal of Hepatology 67:471–479. https://doi.org/10.1016/j.jhep.2017.04.002
